# Oxide Ion Conductivity, Proton Conductivity, and Phase Transitions in Perovskite-Derived Ba_3–*x*_Sr_*x*_YGa_2_O_7.5_ 0 ≤ *x* ≤ 3 Materials

**DOI:** 10.1021/acs.chemmater.1c04372

**Published:** 2022-03-28

**Authors:** Chloe A. Fuller, James Iain Murrell, Douglas A. Blom, Thomas Vogt, Weiguo Zhang, P. Shiv Halasyamani, Ivana Radosavljevic Evans, John S. O. Evans

**Affiliations:** † Department of Chemistry, Durham University, Science Site, South Road, Durham DH1 3LE, United Kingdom; ‡ Department of Chemical Engineering and NanoCenter, University of South Carolina, Columbia, South Carolina 29208, United States; § Department of Chemical Engineering, Chemistry and Biochemistry and NanoCenter, University of South Carolina, Columbia, South Carolina 29208, United States; ∥ Department of Chemistry, University of Houston, Houston, Texas 77204-5003, United States

## Abstract

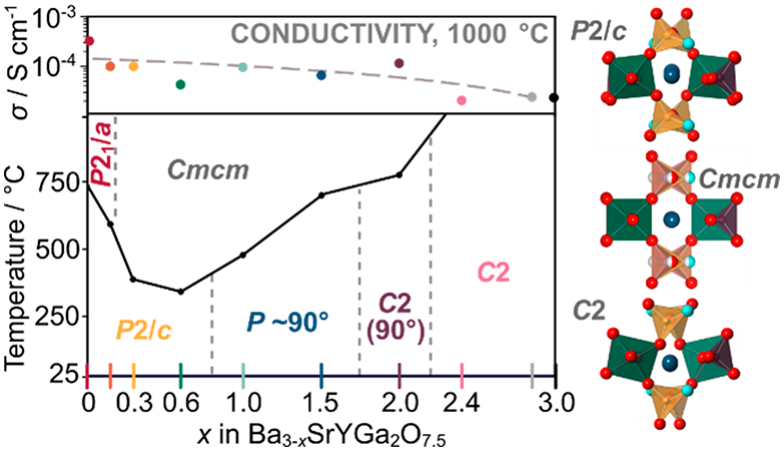

We report the synthesis, structural characterization, and oxide ion and proton conductivities of the perovskite-related Ba_3–*x*_Sr_*x*_YGa_2_O_7.5_ family. Single-phase samples are prepared for 0 ≤ *x* ≤ 3 and show a complex structural evolution from *P*2/*c* to *C*2 space groups with an increase in *x*. For 1.0 ≲ *x* ≲ 2.4, average structures determined by X-ray and neutron powder diffraction show metrically orthorhombic unit cells, but HAADF-STEM imaging reveals this is caused by microstructural effects due to intergrowths of the Ba- and Sr-rich structure types. Variable-temperature powder diffraction studies suggest that 0 ≲ *x* ≲ 2.4 compositions undergo a phase transition upon being heated to space group *Cmcm* that involves disordering of the oxygen substructure. Thermal expansion coefficients are reported for the series. Complex impedance studies show that the Ba-rich samples are mixed proton and oxide ion conductors under moist atmospheres but are predominantly oxide ion conductors at high temperatures or under dry atmospheres. Sr-rich samples show significantly less water uptake and appear to be predominantly oxide ion conductors under the conditions studied.

## Introduction

Materials displaying either proton or oxide ion mobility are crucial for several important applications: as electrolytes in fuel cells, in sensors, and as ion-permeable membranes.^1−3^ There is significant interest in identifying new materials with high conductivity at low temperatures. Structural families of interest for oxide ion conductivity include fluorite derivatives,^4−6^ apatites,^7−9^ melilites,^10−13^ La_2_Mo_2_O_9_,^14,15^ and others. Perovskite derivatives such as ABO_3−δ_ and brownmillerites A_2_B_2_O_5_ are of interest for both oxide ion and proton conductivity.

**Figure 1:**
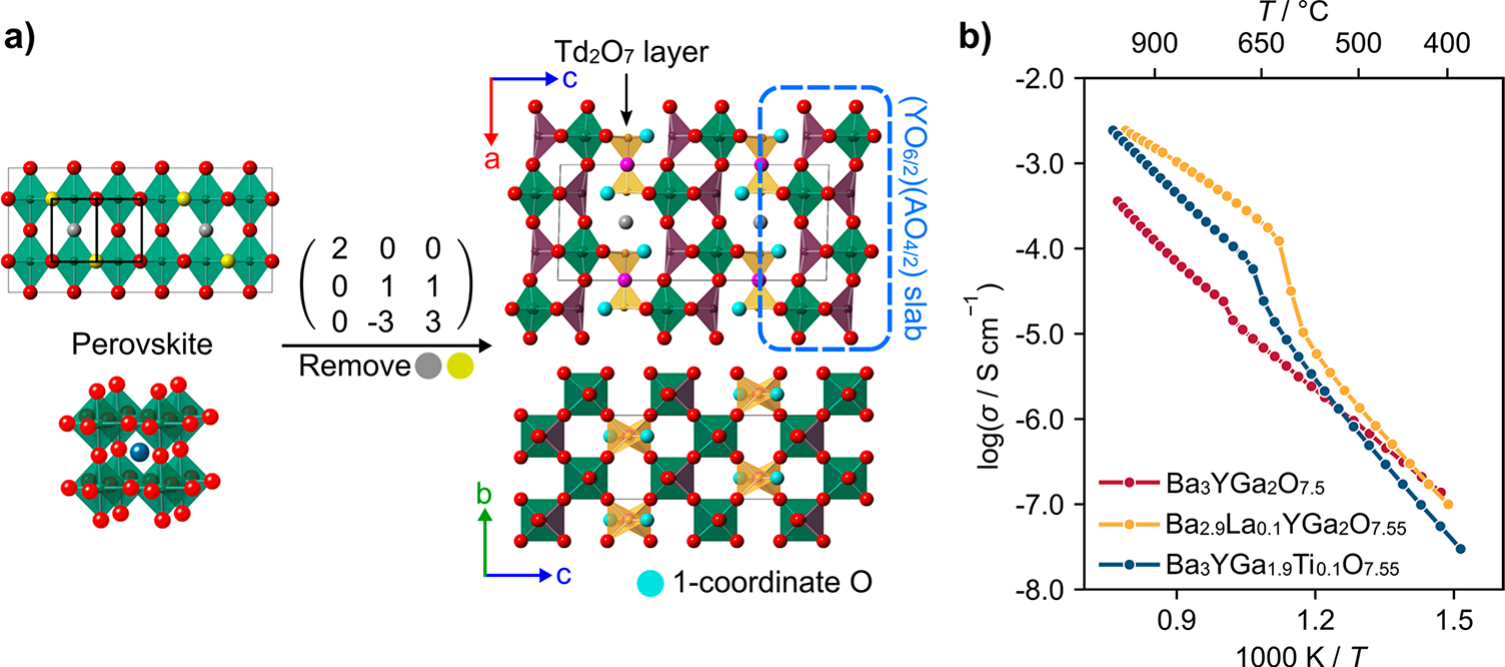
(a) Relationship of perovskite to A_3_OhTd_2_O_7.5_ structure type. One coordinate oxygen is colored cyan. Gray shows the vacant oxygen site discussed in the text. The Td–O–Td oxygen is colored pink. (b) Conductivity of Ba_3_YGa_2_O_7.5_ and two substituted derivatives.

In recent work, we have shown high proton and oxide ion conductivity in a family of perovskite-related compounds with composition A_3_OhTd_2_O_7.5_, where A is a 2+ cation and Oh and Td are octahedrally and tetrahedrally coordinated 3+ cations, respectively (Figure 1).^16^ Relative to perovskite, the formula can be expressed as ABO_3−δ_ with B = Oh_1/3_Td_2/3_ and δ = 0.5. This family also has a close relationship to brownmillerites, A_2_B_2_O_5_, which can be expressed as ABO_2.5_ with B = (Oh_1/2_Td_1/2_). The formal relationship to perovskite is shown in Figure 1a, where A sites have been omitted for the sake of clarity. Here two oxygen sites (six per cell in total) are removed from a cell with *V* = 12*V*_perovskite_ created by the transformation [(2, 0, 0), (0, 1, 1), (0, −3, 3)]. Subsequent oxygen shifts and polyhedral rotations lead to the A_3_OhTd_2_O_7.5_ topology that has an interrupted network built from corner-sharing octahedra and tetrahedra.^17^ The structure can be described as containing layers two polyhedra wide of fully connected _∞_^2^[(OhO_6/2_)(TdO_4/2_)]^4–^ alternating with layers containing Ga_2_O_7_ groups.

One key feature of this structure is that two corners of the Ga_2_O_7_ tetrahedra are not shared with other framework polyhedra, so the Niggli description becomes A_3_(OhO_6/2_)(TdO_4/2_)(TdO_3/2_O). This framework breaking is a natural consequence of the high Td content. A second interesting feature of the Td_2_O_7_ layers is that they show an obvious potential mechanism for oxygen positional disorder via a correlated motion of TdO_3/2_O groups. Similar motions have been shown to enable high ion mobility in several materials.^4,10,14,18−35^ For example, one can imagine the pink Td–O–Td oxygen on the right of Figure 1a moving to the unoccupied gray site. This would cause a net shift of Td_2_O_7_ groups by half of a unit cell vertically. Locally it could occur by coupled rotations of the underlinked TdO_3/2_O tetrahedra. We have previously shown^16^ that this type of transition occurs in alternate Ga_2_O_7_ layers at high temperatures in Ba_3_YGa_2_O_7.5_, leading to a change in Td_2_O_7_ ordering from the “Abakumov type”^17^ to the “Tamazyan type”.^36^ We have also shown that it has implications on high-temperature ionic conductivity, with a significant increase in conductivity around the phase transition (Figure 1b). Abakumov et al.^17^ also showed that defects of this type can occur in Ba_3_DyAl_2_O_7.5_.

In fact, oxygen disorder leading to 50% occupancy of the pink and gray sites would give rise to disordered chains of TdO_3/2_O tetrahedra and space group symmetry *Amma*, or *Cmcm* in the standard setting. This is a useful aristotype for exploring the structures of experimentally observed phases. Oxygen ordering on the gray and pink sites of Figure 1 from this aristotype leads to specific patterns of Td_2_O_7_ between the (OhO_6/2_)(TdO_4/2_) slabs. Coupled rotations of the octahedra and tetrahedra then allow A cations to optimize their bonding and cause the Td_2_O_7_ groups to distort from the staggered configuration of Figure 1 (with a torsion angle between the one-coordinate oxygens of 180°) to the almost eclipsed configurations discussed below.

In this paper, we explore the structural chemistry and physical properties of the new series of compounds Ba_3–*x*_Sr_*x*_YGa_2_O_7.5_ with 0 ≤ *x* ≤ 3, where we observe a family rich in structural transitions as a function of both composition and temperature. The average structure across the series is studied using powder X-ray and neutron diffraction data, and the local structure and defects are probed using HAADF-STEM imaging. Impedance spectroscopy has been used to measure the oxide and proton conductivity across the series, and the properties are correlated with structural changes.

## Experimental Section

### Synthesis

Two gram polycrystalline samples of the Ba_3–*x*_Sr_*x*_YGa_2_O_7.5_ series (where *x* = 0.0, 0.15, 0.3, 0.6, 1.0, 1.5, 2.0, 2.4, 2.85, and 3) were synthesized from stoichiometric amounts of BaCO_3_ (Fisher Scientific, 99%), SrCO_3_ (Sigma-Aldrich, ≥99.9%), Y_2_O_3_ (Sigma-Aldrich, 99.99%), and Ga_2_O_3_ (Sigma-Aldrich, ≥99.99%). Starting compounds were ground together in ethanol, pelletized, and heated in alumina crucibles at 1200 °C for 12–18 h. Samples were reground, repelletized, and reheated until no changes in the powder X-ray diffraction pattern occurred. Total heating times amounted to roughly 150 h at 1200 °C. All of the prepared phases formed white powders.

### X-ray Diffraction

A Bruker D8 Advance diffractometer in Bragg–Brentano geometry with Cu Kα radiation and a Lynx-eye PSD detector was used for all X-ray powder diffraction measurements. A 2θ range of 10–120° and collection times of ≤6 h were used for final patterns, and an internal Si standard [*a* = 5.431195(9) Å at room temperature] was used for accurate cell parameter determination. Analysis of the diffraction data was performed using the Rietveld method^37^ implemented in the Topas Academic software.^38,39^ Structures were drawn using Crystalmaker or Vesta.^40^

Variable-temperature X-ray diffraction patterns were recorded with an Anton Paar HTK1200 furnace attachment from 25 to 1000 °C. Furnace temperatures were calibrated on the basis of an external Al_2_O_3_ standard.^41^ Diffraction data were measured for the 2θ range of 10–120° in 0.02° steps every 20 °C on heating and cooling for between 20 min and 3 h, depending on the quality required. Such experiments were performed for all single-phase samples. All resulting diffraction patterns were analyzed by sequential Rietveld fitting in Topas Academic.^38,39^

### Neutron Diffraction

Ten gram powdered samples of Sr_3_YGa_2_O_7.5_ and Ba_1.5_Sr_1.5_YGa_2_O_7.5_ were synthesized for neutron scattering experiments. Once phase purity was achieved, the samples were dried at 1200 °C overnight and then quench-cooled to room temperature. Then, ∼6 g of each sample was then immediately packed into sealed 8 mm diameter vanadium cans. High-quality data sets were acquired for each sample at room temperature for 8 h each on the POLARIS instrument of the Rutherford Appleton Laboratory. Bragg data were extracted using the established analysis routines in Mantid.^42^ All structural analyses were performed using Topas Academic.^38,39^

Rietveld fits were performed using the three highest-resolution data banks (banks 3–5), covering a total *d* spacing range of 0.15–6.0 Å.

### Scanning Transmission Electron Microscopy

AC-STEM data in Z-contrast HAADF imaging mode were collected from powdered samples of Ba_3_YGa_2_O_7.5_, Ba_1.5_Sr_1.5_YGa_2_O_7.5_, BaSr_2_YGa_2_O_7.5_, and Sr_3_YGa_2_O_7.5_. The powders were lightly ground in an agate mortar and pestle before being quickly loaded onto holey carbon-coated Cu TEM grids. A JEOL JEM2100F instrument with a CEOS aberration corrector for the electron probe was used at 200 kV to image the sample. The probe-forming aperture semiangle was 17.5 mrad, and the Fischione model 3000 ADF detector spanned 75–300 mrad. Images were acquired synchronously with ac electric power (60 Hz) to minimize 60 Hz artifacts in the images. A pixel dwell time of 15.7 μs was used throughout. The x-scan direction of the instrument was adjusted to be parallel to the long axis of the unit cells.

### Thermogravimetric Analysis

Mass changes on heating and cooling cycles were measured using a PerkinElmer TGA 8000 instrument. In one experiment, small, powdered samples of Ba_3_YGa_2_O_7.5_, Ba_1.5_Sr_1.5_YGa_2_O_7.5_, and Sr_3_YGa_2_O_7.5_ were left exposed to the laboratory air in open glass vials for 3 days before TGA was performed on heating from 30 to 1000 °C and cooling back to ambient temperature at a rate of 10 °C min^–1^ under flowing atmospheric air. In a second experiment, *x* = 0.0, 0.3, 1.0, 1.5, 2.0, and 3.0 samples were heated to 1150 °C, cooled at a rate of 5 °C per min^–1^ to 350 °C, removed from the furnace, and sealed in vials within 20 min.

### Solid State ^1^H Nuclear Magnetic Resonance (NMR)

The hydration behavior of Sr_3_YGa_2_O_7.5_ and Ba_1.5_Sr_1.5_YGa_2_O_7.5_ was investigated using ^1^H NMR. Each sample was heated to 1200 °C for 12 h and then quench-cooled to room temperature. ^1^H NMR spectra were recorded immediately on a Bruker Avance III HD spectrometer with tetramethylsilane as a reference. Samples were then placed in a 74% relative humidity chamber for 1 week, and the proton spectra were re-collected under the same conditions.

### Second-Harmonic Generation (SHG) Measurements

Samples of Ba_3–*x*_Sr_*x*_YGa_2_O_7.5_ (*x* = 0.15, 1.5, 2, and 2.85) were placed in fused silica tubes with a diameter of 4 mm. A 1064 nm pulsed Nd:YAG laser (Quantel Laser, Ultra 50) was used to generate the fundamental light, and the SHG intensity was recorded at room temperature by a digital phosphor oscilloscope (Tektronix, TDS3032). Intensity comparisons were made with the known SHG material α-SiO_2_ under the same conditions.^43^

### Impedance Spectroscopy

Powders for impedance measurements were uniaxially pressed into 10 mm pellets and sintered. Each pellet was coated in platinum ink and mounted on a Probostat A-6 cell. The electrodes were set by heating at 1000 °C for 1 h. A Solartron 1260 frequency-response analyzer was used to measure and record impedance spectra approximately every 20 °C. Frequencies of 0.1–10^7^ Hz and voltages of 100–1000 mV were used for the measurements, and results were analyzed in ZView (Scribner Associates). For standard measurements, the impedance was recorded on cooling at a rate of 2 °C min^–1^ from 1000 °C to room temperature in air. Where necessary, further measurements were performed on heating back to 1000 °C. Impedance was also measured under dry flowing N_2_ for most of the series and recorded over multiple heating and cooling cycles to ensure equilibrium had been achieved. Laboratory access during COVID-19 lockdowns meant that two samples were not measured under N_2_; we assume their properties can be interpolated from adjacent compositions.

## Results and Discussion

**Figure 2:**
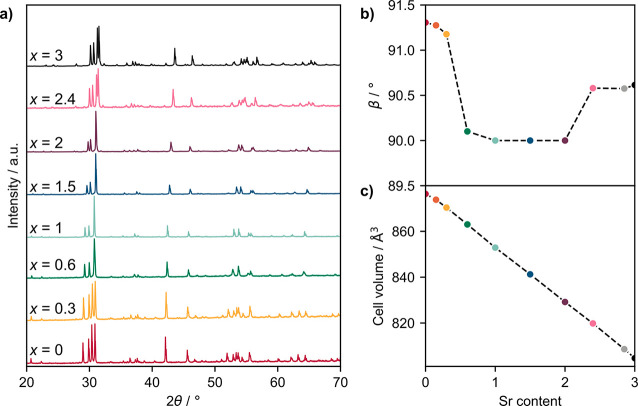
(a) Diffraction patterns of Ba_3–*x*_Sr_*x*_YGa_2_O_7.5_ along with the evolution of (b) cell angle β and (c) cell volume with Sr content. In panels b and c, standard uncertainties are smaller than the size of the points plotted.

Ten Ba_3–*x*_Sr_*x*_YGa_2_O_7.5_ compounds (0 *≤ x* ≤ 3) were successfully prepared via a solid state route. All compounds were confirmed to be essentially single phase (≳99%) by powder diffraction; their diffraction patterns and selected cell parameters are shown in Figure 2 and Table 1. Further unit cell parameter plots are given in Figure S1.

**Table 1:** Ba_3–*x*_Sr_*x*_YGa_2_O_7.5_ Unit Cell Parameters

Sr content (*x*)	*a* (Å)	*b* (Å)	*c* (Å)	β (deg)	volume (Å^3^)
0	7.94977(5)	5.97099(4)	18.4659(2)	91.3061(6)	876.31(1)
0.15	7.94479(8)	5.96656(7)	18.4373(2)	91.2789(7)	873.76(2)
0.3	7.94092(5)	5.96366(5)	18.4209(2)	91.1880(6)	872.17(1)
0.6	7.92035(9)	5.95458(7)	18.3021(3)	90.101(1)	863.17(2)
1	7.89502(9)	5.93842(7)	18.1837(2)	89.997(5)	852.52(2)
1.5	7.87200(7)	5.91962(6)	18.0748(2)	89.998(4)	842.27(2)
2^a^	7.85705(9)	5.88988(6)	17.8977(2)	90.013(5)	828.24(2)
2.4^a^	7.8445(2)	5.8543(1)	17.8454(4)	90.637(2)	819.48(3)
2.85^a^	7.8192(1)	5.8227(1)	17.7519(3)	90.614(1)	808.18(2)
3^a^	7.81193(9)	5.81179(6)	17.7213(2)	90.6164(7)	804.52(1)

a *a* and *c* values swapped with *A* centering to match the Abakumov cell with *c* > *a* > *b*.

**Figure 3:**
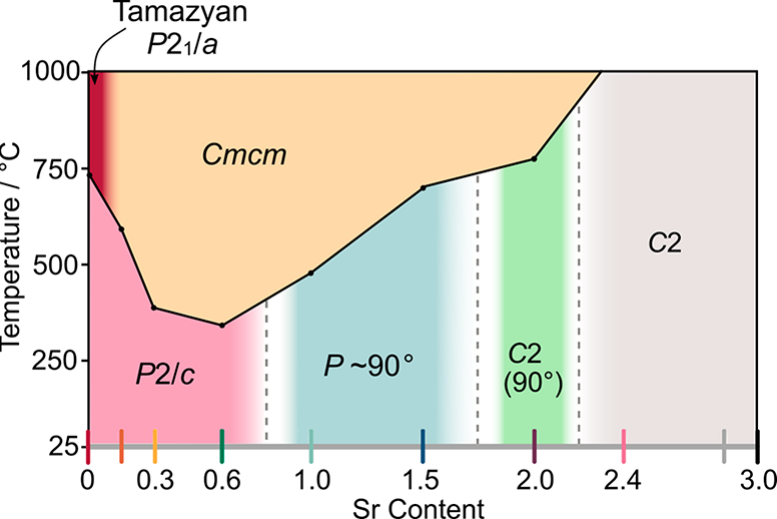
Phase diagram of Ba_3–*x*_Sr_*x*_YGa_2_O_7.5_. Solid black lines join points representing measured transition temperatures, and dashed gray lines indicate approximate boundaries between the compositions studied.

The cell volume decreases linearly with Sr content in line with Vegard’s law, but cell angle β suggests significant structural changes across the series, with values suggesting both monoclinic and orthorhombic structures exist. The structural evolution was investigated using a combination of powder X-ray and neutron diffraction, HAADF-STEM, and SHG as a function of composition and temperature to produce the phase diagram shown in Figure 3. The following sections will discuss each structure type at room temperature and higher temperatures in turn.

### Room-Temperature Structures

**Figure 4:**
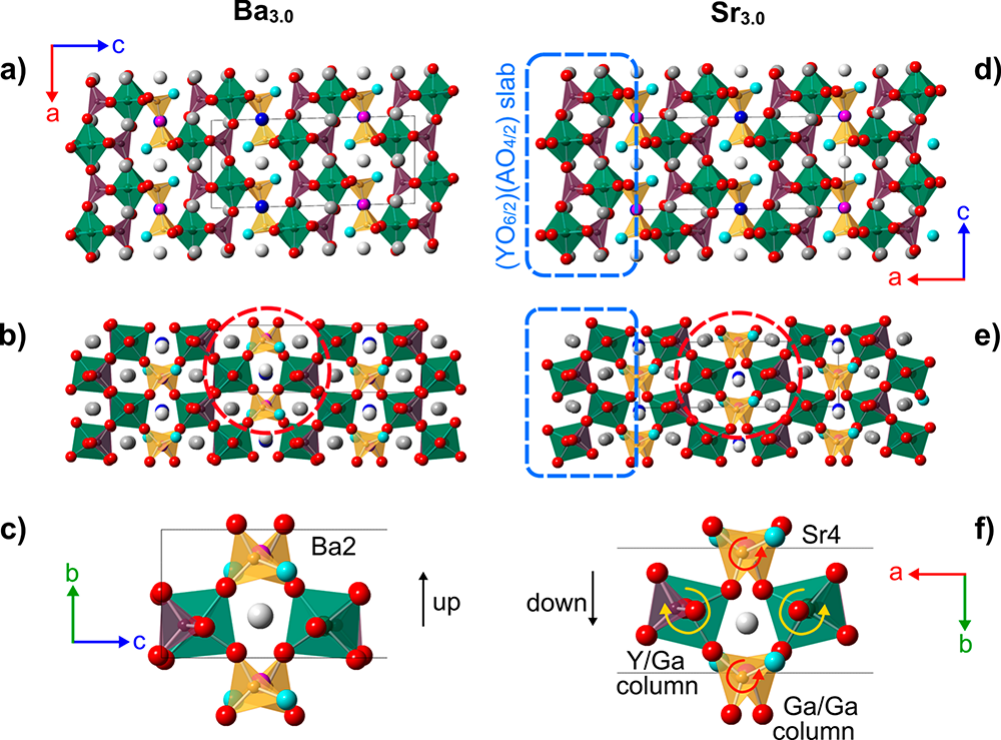
Abakumov *P*2/*c* and Wang *C*2 structures of Ba_3_YGa_2_O_7.5_ and Sr_3_YGa_2_O_7.5_ viewed perpendicular to and down Ga_2_O_7_ axes. Ba2/Sr4 coordination environments highlighted by red circles in panels b and e are enlarged in panels c and f, respectively.

The structure of end member Ba_3_YGa_2_O_7.5_ has been discussed in detail elsewhere^16^ and adopts an Abakumov-type^17^*P*2/*c* structure at room temperature. Views down different axes are shown in panels a and b of Figure 4. Compositions Ba_2.85_Sr_0.15_YGa_2_O_7.5_ and Ba_2.7_Sr_0.3_YGa_2_O_7.5_ were both found to be isostructural with Ba_3_YGa_2_O_7.5_ with a similar β of ∼91.3°. Rietveld plots are given in Figure S2. This is consistent with SHG measurements on Ba_2.85_Sr_0.15_YGa_2_O_7.5_ that showed no response, as expected for a centrosymmetric structure. On the basis of observations across the series, we assume that Sr and Ba cations are distributed randomly.

X-ray diffraction patterns for *x* = 0.6, 1.0, and 1.5 are different from the *x* ≤ 0.3 Ba-rich compositions, with just three strong peaks visible around a 2θ of 30° instead of four. The monoclinic *P*2/*c* model provides a good fit to the *x* = 0.6 pattern, with all peaks indexed and β being refined to 90.101(1)°, significantly smaller than those of lower-*x* compositions. As more Sr is added, the fit becomes worse for certain peaks (marked by arrows in Figure S3) whose intensities are increasingly overcalculated by the *P*2/*c* model. The monoclinic angles for compositions Ba_2_SrYGa_2_O_7.5_ and Ba_1.5_Sr_1.5_YGa_2_O_7.5_ refine to 89.997(5)° and 89.998(4)°, respectively, suggesting a change to orthorhombic symmetry. The prediction of weak peaks that are not observed experimentally shows that the *P*2/*c* model does not properly describe these structures.

Room-temperature neutron powder diffraction data of Ba_1.5_Sr_1.5_YGa_2_O_7.5_ were collected on POLARIS for 8 h and confirmed a metrically orthorhombic cell. The highest-symmetry *Cmcm* orthorhombic model of Figure 1 gave a reasonable approximation of the X-ray and neutron patterns, but the intensities of several neutron peaks were over- or undercalculated and some not predicted at all, even with all atomic positions being refined and all atoms allowed to have anisotropic ADPs. In an attempt to find the correct space group, we analyzed all possible subgroups of the *Cmcm* parent down to a *P*1 child base structure using the ISODISTORT tools^44−46^ and the method described previously for Bi_2_Sn_2_O_7_.^47^ The 67 subgroups tested and their relationships are shown graphically in Figure S4 as a subgroup tree. For each, the amplitudes of the appropriate distortion modes were refined against the bank 4 neutron diffraction data. Occupational distortion modes were not considered, so all models contained disordered Ga_2_O_7_ chains. The final refined models were ranked by quality of fit (*R*_wp_) and the number of structural parameters.

The best model identified by this process was the monoclinic *P*2/*c* model [subgroup 47 (see Figure S4)] with a β angle very close to 90.0° [refined value of 88.997(4)°]. This has 47 structural parameters and an *R*_wp_ of 4.4%. However, as discussed above, this *P*2/*c* model does not fit the data perfectly. To improve the fit any further, the number of parameters needed to almost double to 91, and even the *P*1 model [with 177 structural parameters (Figures S5 and S6)] did not fully explain all of the diffraction features observed.

Some insight into why none of the simple average structural models fully explains the diffraction data can be gained by examining the weak peaks that violate cell centering [*A* centering in the setting used (see the arrows in Figure S7)]. These *P*-only peaks are found to be significantly broader than others, suggesting only short-range order. They were also not visible in the laboratory X-ray data, suggesting a significant oxygen or lighter atom contribution. We could not identify any model that simultaneously described the *P*-only neutron peaks without significant overcalculation of their X-ray intensity. We therefore also investigated structures with different Ga_2_O_7_ ordering patterns, including the Tamazyan-type ordering identified for Ba_3_YGa_2_O_7.5_ at high temperatures.^16^ None of these gave a significant improvement in fit. Because average crystallographic methods failed, the structure was further investigated using HAADF-STEM imaging as discussed in the next section.

As the Sr content increases to *x* = 2 (BaSr_2_YGa_2_O_7.5_), the structure remains at least metrically orthorhombic, but diffraction data can no longer be fitted by *P*2/*c* models. Inspection of the X-ray diffraction pattern reveals that *k* + *l* = odd reflections are not present, and thus, the unit cell is centered. However, the *Amma*/*Cmcm* aristotype structure of Figure 1 could not account for all of the peaks. A related, lower-symmetry space group is the monoclinic *C*2 structure used by Wang et al. for Sr_3_YAl_2_O_7.5_.^48^ This gave a much better fit, with all observed peaks accounted for and β being refined to 90.015(4)° (Figure S8). The most appropriate space group for BaSr_2_YGa_2_O_7.5_ might therefore be a subgroup of *Cmcm* and a supergroup of *C*2. The possible orthorhombic space groups *Ama*2, *Amm*2, and *C*222_1_ and monoclinic groups *C*2/*c* and *C*2/*m* were identified from the subgroup tree in Figure S4. BaSr_2_YGa_2_O_7.5_ was found to be SHG-active and thus has a noncentrosymmetric structure. *C*2/*c*, *C*2/*m*, and *Cmcm* can therefore be ruled out because they are centrosymmetric. Models in space groups *Amm*2 and *C*222_1_ fail to fit several reflections in the XRD pattern, whereas *Ama*2 and *C*2 give much better and essentially indistinguishable fits (see Figure S8) and plausible structural models. The *Ama*2 model requires the Ga_2_O_7_ groups to be disordered. While this could occur (e.g., due to an intergrowth of Abakumov- and Tamazyan-type Ga_2_O_7_ ordering), the low-temperature ordered structures of other compositions in the family suggest at least local order is more likely. We therefore favor an ordered *C*2 monoclinic structure with a β very close to 90° at room temperature. The final *C*2 model was refined against a 6 h XRD pattern with Ba and Sr distributed randomly across all A sites and is reported in Table S1.

The *x* ≥ 2.4 Ba_0.6_Sr_2.4_YGa_2_O_7.5_, Ba_0.15_Sr_2.85_YGa_2_O_7.5_, and Sr_3_YGa_2_O_7.5_ compounds were all found to be isostructural, with Wang’s Sr_3_YAl_2_O_7.5_ compound in space group *C*2.^48^ This model was refined against X-ray and neutron diffraction data of Sr_3_YGa_2_O_7.5_ to give the final fits shown in Figure S10 and the coordinates listed in Table S2. Views down different axes are shown in panels d and e of Figure 4. The assignment of space group *C*2 is supported by SHG measurements on Ba_0.15_Sr_2.85_YGa_2_O_7.5_, where a positive signal indicated a noncentrosymmetric structure.

### Room-Temperature HAADF-STEM Imaging

HAADF-STEM imaging was performed on *x* = 0.0, 1.5, 2.0, and 3.0 samples to confirm the structural conclusions described above and gain more insight into local disorder, particularly for Ba_1.5_Sr_1.5_. The heavy atom columns in these structures emphasized in panels b and e of Figure 4 mean that the optimal imaging axis is along the doubled perovskite *a*-axis of the Abakumov structure (equivalent to the *c*-axis of the Wang *C*2 Sr_3.0_ structure). We will use the Abakumov cell setting throughout this discussion; the Wang model would have space group *A*2 in this setting. Because there is no unique way of matching labels between chemically similar sites of these two models, we use the Abakumov numbering scheme and refer to Wang labels where needed.

From panels c and f of Figure 4, it is clear that the key difference between the two structures can be understood in terms of rotations of Ga_2_O_7_ groups around the Ga–O–Ga vector between adjacent (OhO_6/2_)(TdO_4/2_) two-dimensional slabs and the impact this has on the relationship between polyhedral tilts in adjacent slabs. In the Sr_3.0_ structure of Figure 4e, the cell-centering means that Ga_2_O_7_ groups in sequential layers from left to right are related by translational symmetry such that all of the cyan “terminal” oxygens of the closest tetrahedron are oriented to the top right. In contrast, for the primitive Ba_3.0_ structure (Figure 4b), the Ga_2_O_7_’s have their terminal oxygen pointing alternatively to the bottom right for Ga_2_O_7_ groups at *z* ≈ 0.25 and the top right for those at *z* ≈ 0.75. We can at least conceptually imagine the transformation between the two structure types as occurring via a 60° counterclockwise rotation of each of the closer GaO_3/2_O tetrahedra in the view of Figure 4c and a 60° clockwise rotation of the more distant GaO_3/2_O. This goes from the eclipsed Ga_2_O_7_ configuration of Figure 4c via the staggered configuration shown in Figure 1 to the eclipsed configuration of Figure 4f and requires coupled rotations of the corner-linked YO_6/2_ octahedra. This is likely to be a facile local structural distortion due to the underconnected terminal oxygen. The movement of individual atoms required for the transition between the two structures can be visualized by transforming both structures to their common *P*2 subgroup. The Supporting Information contains an animation of the structural changes in ISOVIZ format and can be visualized using the tools of the ISODISTORT software suite of Campbell and Stokes.^44^

**Figure 5:**
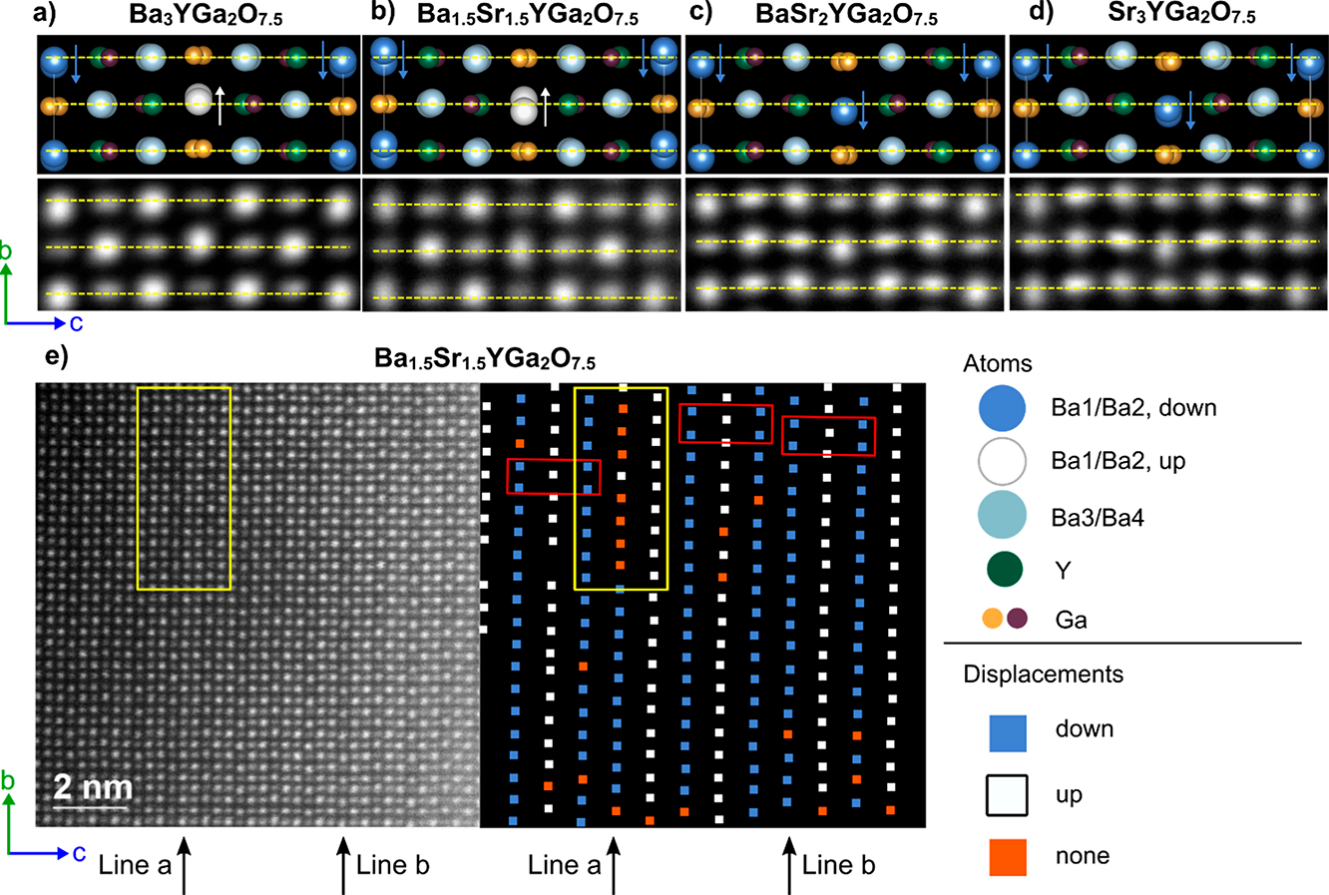
(a–d) STEM images of Ba_3–*x*_Sr_*x*_YGa_2_O_7.5_ (*x* = 0, 1.5, 2, and 3, respectively) with the corresponding structural model from Rietveld refinement. Images are averaged from 106, 66, 104, and 108 unit cells, respectively. Dashed yellow lines are guides to the eye and highlight the displacements of Ba1/Ba2 along the vertical axis. (e) Larger section of a STEM image of Ba_1.5_Sr_1.5_YGa_2_O_7.5_ and schematic of the corresponding Ba1/Ba2 displacements. Red boxes indicate areas of *P*2/*c*-type displacement ordering. Black arrows highlight unusually dim columns of Ba1/Ba2 that could be Sr-rich defects. Yellow boxes highlight a faulted region.

This structural change impacts the nearby A site coordination environments, especially those of A sites lying within the Ga_2_O_7_ layers. In particular, Ba2 (equivalent to Wang’s Sr4) is bonded to two GaO_3/2_O terminal oxygens and four oxygens that these Ga_3/2_O tetrahedra share with YO_6/2_ octahedra that must tilt cooperatively as the tetrahedra rotate. This means that the Ba2 position along the *b*-axis is anticorrelated with the terminal O position: when the terminal oxygen points “up” in Figure 4b, the Ba2 *y* coordinate becomes smaller and shifts “down” and vice versa. Much smaller shifts are observed for the other A site (Ba1) in the Ga_2_O_7_ layers. The pattern of A site shifts observed for the end member structures is then as shown in panels a and d of Figure 5, where the Ba1/Ba2 (blue or white) projected columns have shifts relative to the horizontal yellow dashed lines of down, up, down (↓↑↓) for Ba_3.0_ and down, down, down (↓↓↓) for Sr_3.0_ along the long axis.

The lower portions of panels a and d of Figure 5 show HAADF-STEM images taken from crystallites in this orientation. The bright parts of the image correspond to columns of atoms. The brightest columns are the high-*Z* A atoms (Ba or Sr), and the lighter ones correspond to either Ga/Ga in columns of Ga_2_O_7_ groups or Y/Ga columns in the (YO_6/2_)(GaO_4/2_) slabs. In each case, the image quality has been enhanced by averaging over multiple equivalent parts of a larger-area image (such as that shown in Figure 5e). In both compounds, we see that the Ga/Ga and Y/Ga columns appear as ellipses elongated parallel to the *c*-axis. This reflects an offset in the individual atom *z* coordinates of ∼0.6 Å in projection. Y/Ga columns are more intense than Ga/Ga columns due to both the higher atomic number of Y and the larger number of oxygens in the column. We also see a larger tilt of the ellipse principal axis of Ga/Y columns relative to the *c*-axis in the Sr_3.0_ case, consistent with the larger offsets of Y/Ga on the *b*-axis (∼0.06 and 0.02 Å for Sr_3.0_ and Ba_3.0_, respectively). The principal axes of Ga/Ga ellipses are constrained by symmetry to lie along the *c*-axis.

The Ba3/Ba4 columns inside the (YO_6/2_)(GaO_4/2_) slabs appear as essentially circular features centered on the horizontal yellow dashed lines. Finally, we see that the Ba1/Ba2 columns (Wang’s Sr3/Sr4) appear elongated parallel to the *b*-axis and show characteristic shifts of ↓↑↓ relative to the yellow dashed line for the Ba_3_ case and ↓↓↓ for Sr_3.0_. The local structure is therefore in full agreement with the powder crystallography.

Panels b and c of Figure 5 show equivalent projected structural models and images for intermediate compositions Ba_1.5_Sr_1.5_ and Ba_1.0_Sr_2.0_, respectively. For *x* = 2 Ba_1.0_Sr_2.0_, the powder crystallography and positive SHG signal favored a monoclinic *C*2 model with a β of ≈90° over a *Cmcm* orthorhombic model. The STEM image shows excellent agreement with this model. The elongated shapes expected for Ga/Ga and Y/Ga columns are well reproduced. A3/A4 columns appear circular and are at *y* values similar to those of the Ga/Ga and Y/Ga columns, while the A1/A2 columns show a characteristic ↓↓↓ shift, the magnitude of which is slightly smaller than that for Sr_3.0_. We note that no shifts of A1/A2 columns would be expected in the *Cmcm* model.

**Figure 6:**
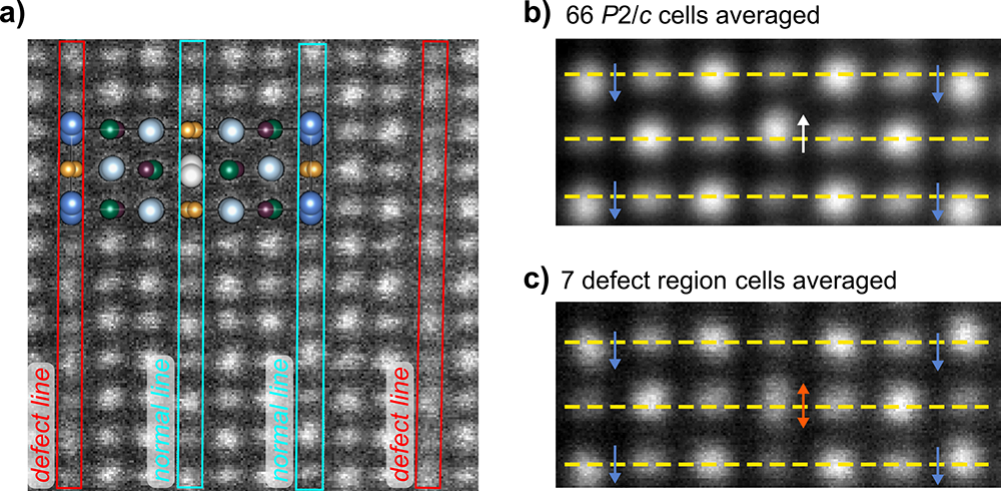
(a) Area of STEM image highlighting defect layers in Ba_1.5_Sr_1.5_YGa_2_O_7.5_. (b and c) Averaged images from unfaulted regions and the faulted regions highlighted in the yellow box of Figure 5e, respectively.

Panels b and e of Figure 5 show images of Ba_1.5_Sr_1.5_ for which the best Rietveld fit failed to perfectly reproduce some of the weak diffraction features, and our favored model was *P*2/*c* with a β of ∼90°. An averaged image from 66 unit cells is shown in Figure 5b, and we see the characteristic ↓↑↓ A1/A2 shift pattern of the Ba_3.0_ composition, supporting the primitive space group choice. The offsets also rule out an alternative orthorhombic *Pmca* model in which no displacements would be observed. Figure 5e shows an image of a larger area corresponding to ∼6.5 unit cells in *c* and ∼20 unit cells in *b*. This larger area shows that there is significant faulting in the A1/A2 displacements. On the right of Figure 5e, we have color-coded the A1/A2 displacements as blue for ↓, white for ↑, and orange where there is no clear displacement direction. No spot is drawn in one point of the image where there was a probe positioning error. The Ba_3.0_*P*2/*c* structure then corresponds to a blue–white–blue alternation between the vertical lines of spots from left to right. This is seen, for example, in the three unit cells outlined in red and occurs over much of the image area. There is, however, clear evidence for defect regions corresponding to Sr_3.0_-type order (↓↓). Lines labeled a and b, for example, show the opposite displacement to that expected on the basis of the line to their left. Interestingly, line a contains a significant number of orange columns where the displacement direction could not be determined. This is presumably related to disorder within those atomic columns as they run into the page. The prevalence of planar defects is highlighted in Figure 6a, where we see vertical lines where the A1/A2 columns are less intense relative to the Ga/Ga columns than elsewhere and show a more diffuse background between the columns. Most of these lines correspond to the zero-shift columns discussed above and are associated with ↓↓ faults. As such, they are likely to be caused by cation segregation giving rise to Sr-rich (lower-*Z*) A-site regions. We also saw evidence for this type of short-range ordering in the broader *P*-only powder diffraction peaks discussed above.

Panels b and c of Figure 6 compare the A1/A2 shifts in ordered regions of the structure with those from the cells highlighted in yellow in Figure 5e, where significant faulting is observed. The middle line of A1/A2 sites is significantly smeared out and is centered (on average) on the dashed yellow line reflecting disorder down the column. An animated version of Figure 5e that emphasizes the A1/A2 shifts is available as a “flip chart movie” of each unit cell area (Supporting Information). While these defects were common in Ba_1.5_Sr_1.5_, they were not observed in Ba_1.0_Sr_2.0_.

### High-Temperature Structures

**Figure 7:**
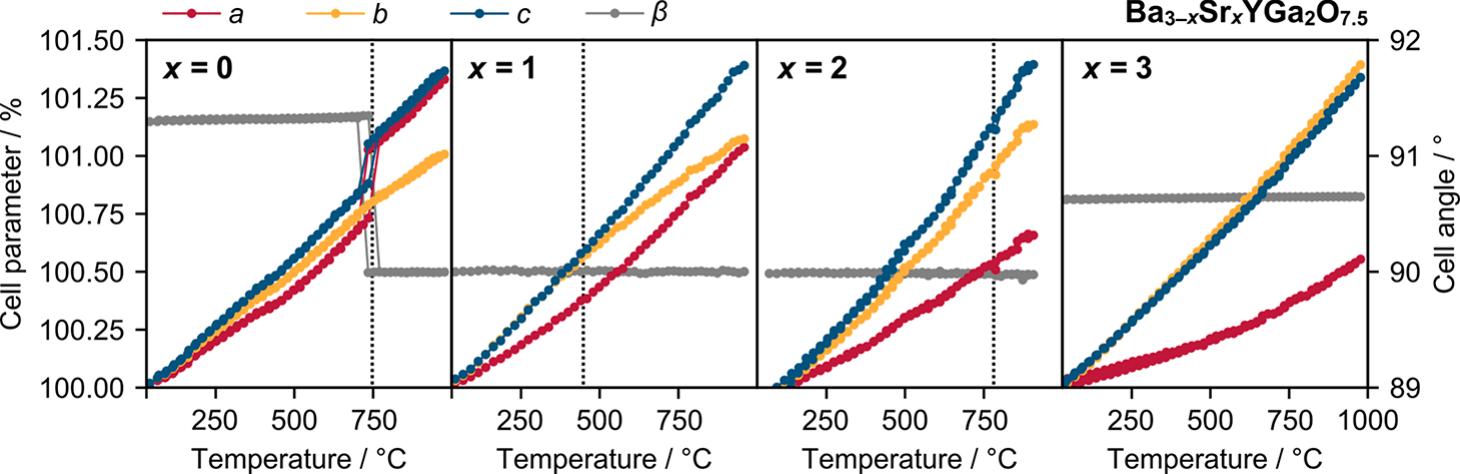
Evolution of the cell parameters with temperature for a selection of Ba_3–*x*_Sr_*x*_YGa_2_O_7.5_ compounds from variable-temperature PXRD. Cell parameters *a*, *b*, and *c* are given as percentages of their room-temperature values on the left-hand axes, relative to the Abakumov cell, and β angles are given on the right-hand axes. Approximate phase transition temperatures are indicated by the vertical dotted lines. Data from both heating and cooling runs are superimposed for *x* = 0, 2, and 3. Standard uncertainties are smaller than the symbol sizes.

Figure 7 shows the evolution of unit cell parameters with temperature for a selection of compositions measured by variable-temperature XRD. Data from both heating and cooling cycles are shown except for those of Ba_2_SrYGa_2_O_7.5_, which showed evidence for water loss on first heating (further information is available in Figures S12 and S13 and Table S3).^16^

Ba_2.85_Sr_0.15_YGa_2_O_7.5_ and Ba_2.7_Sr_0.3_YGa_2_O_7.5_ undergo a phase transition similar to that of Ba_3_YGa_2_O_7.5_ (Figure S11) on heating. The phase transition temperature decreases smoothly with an increase in Sr content. Above the phase transition, the diffraction patterns can be fitted well by the disordered *Cmcm* model of Figure 1. The small peaks that indicated a change in Ga_2_O_7_ ordering in Ba_3_YGa_2_O_7.5_ are not as apparent in either of these compositions. There is, however, some diffuse scattering in similar positions in the high-temperature XRD patterns of both compounds, hinting that some short-range Tamazyan-type ordering may be present (white box in Figure S14). It seems likely that faults in planes perpendicular to the long unit cell axis broaden and weaken these peaks.

For the metrically orthorhombic 0.6 ≤ *x* ≤ 1.5, there are no obvious changes in peak positions or intensities in the raw diffraction data; however, the cell parameter temperature variation suggests a phase transition in all three compounds. In particular, we see a reduction in thermal expansion along the *b*-axis at high temperatures, similar to that observed in Ba_3_YGa_2_O_7.5_. The temperature at which the change occurs increases with Sr content, with *T*_c_ values of 370, 420, and 700 °C for *x* values of 0.6, 1.0 and 1.5, respectively. All of the diffraction patterns across the temperature range were Rietveld-fitted with both *P*2/*c* and *Cmcm* models with atomic positions fixed at the values refined against the Ba_3_YGa_2_O_7.5_ diffraction data at 25 and 1000 °C, respectively; *R*_wp_ is plotted versus temperature in Figure S15. The temperatures at which the *Cmcm* model becomes as good as *P*2/*c* correspond to the proposed phase transition temperatures, suggesting that the structure might undergo a transition to the *Cmcm* model at high temperatures. However, we do not have neutron diffraction data at high temperatures for these compositions, so the nature of this transition cannot be stated definitively.

The *x* = 2 BaSr_2_YGa_2_O_7.5_ compound also has a phase transition, occurring at ∼770 °C on heating and cooling. This phase transition is characterized by the disappearance of four weak peaks in the XRD pattern between 2θ values of 25° and 45° (highlighted by arrows in Figure S16). These are the same peaks that could not be indexed by a *Cmcm* model, suggesting the transition is between the low-temperature *C*2 structure and the high-temperature disordered *Cmcm* structure.

For *x* > 2 compositions, we observe smooth increases in cell parameters as a function of temperature and no evidence of major structural changes. Samples remain monoclinic (β ≈ 90.6°) up to at least 1000 °C.

### Water Uptake

Thermogravimetric analysis was performed on freshly heated samples with *x* values of 0.0, 0.3, 1.0, 1.5, 2.0, and 3.0 on heating and cooling from room temperature to 1000 °C under dry air. Experiments were also performed on *x* = 0.0, 1.5, and 3.0 samples after deliberate exposure to moist atmospheres for an extended period. The results are shown in Figure S17 and supported by the proton NMR spectra in Figure S18. The least basic *x* = 3.0 Sr_3_YGa_2_O_7.5_ shows minimal water uptake upon exposure to atmospheric moisture, with small mass losses consistent with surface adsorption. Ba-containing samples show more significant uptake, with the *x* = 0 composition becoming X-ray amorphous after exposure to moisture for 3 days and showing a 19.5% mass loss on heating. More minor water uptake (∼0.5%) occurs during the ∼4 h period as samples are furnace-cooled from high temperatures. This is consistent with the “humps” observed around 500 °C in the unit cell plots in Figure 7 on both heating and cooling cycles for *x* values of 0 and 2. We note that no such effects are observed for Sr_3_YGa_2_O_7.5_.

### Conductivity

**Figure 8:**
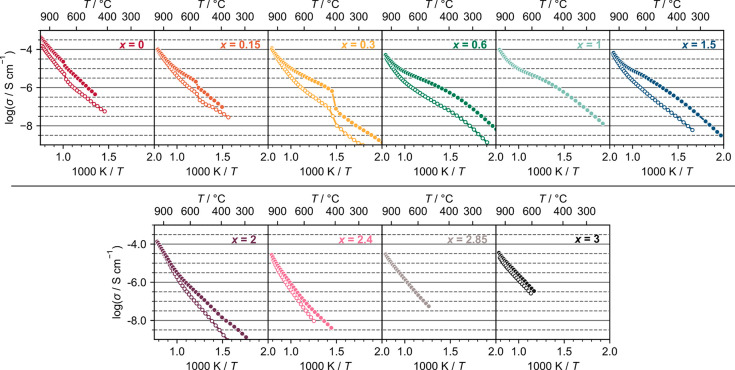
Conductivity data for the Ba_3–*x*_Sr_*x*_YGa_2_O_7.5_ series in air (filled points) and dry N_2_ (empty points). Error bars are smaller than the symbols plotted. Data were measured in air only for *x* = 1 and *x* = 2.85.

**Figure 9:**
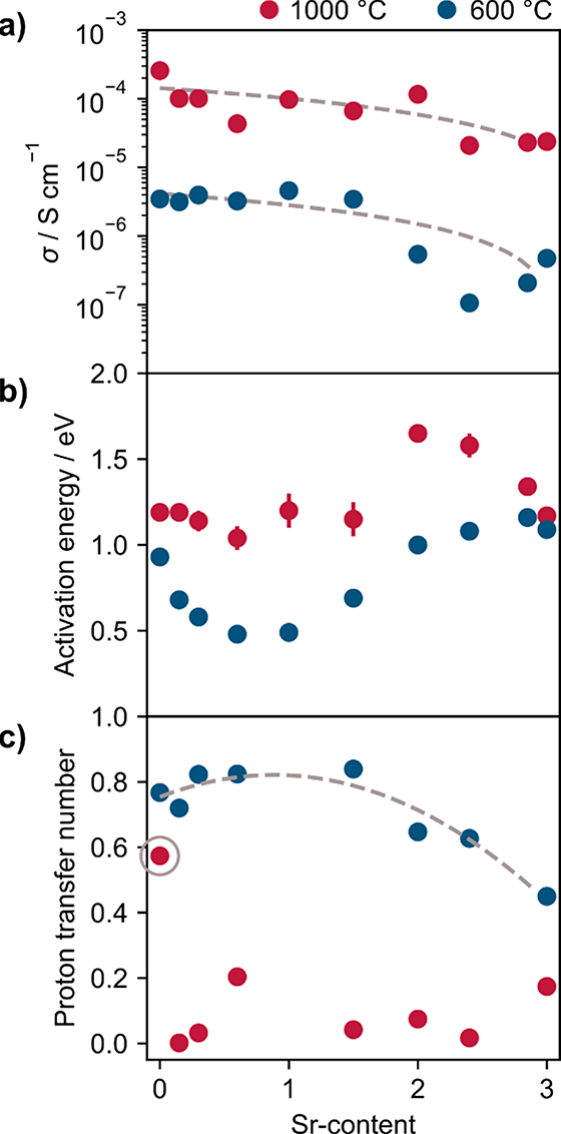
Conductivity trends as a function of Sr content in Ba_3–*x*_Sr_*x*_YGa_2_O_7.5_. (a) Conductivity in air. Gray dashed lines show a linear fit to σ as a guide to the eye. (b) Activation energies in air. (c) Proton transfer numbers. The gray circle highlights Ba_3_YGa_2_O_7.5_, and the dashed line is a binomial fit to the data as a guide to the eye. Error bars are smaller than symbols where they are not plotted.

Impedance spectroscopy was performed in air and dry N_2_ to measure the conductivity of representative compositions. Data for individual compositions are shown in Figure 8, with collated information summarized and plotted as a function of composition in Figure 9.

The Ba-rich *x* ≤ 0.3 *P*2/*c* compounds all show behavior similar to that of Ba_3_YGa_2_O_7.5_. There is a significant conductivity jump at the *P*2/*c* to *Cmcm* phase transition, which becomes more prominent with higher Sr content.

Each composition shows a proton contribution to conductivity at low temperatures, evidenced by the difference in conductivity in air and dry N_2_ at low temperatures, and the low activation energy at low temperatures (Figure 9c). At high temperatures, the conductivity of *x* = 0 Ba_3_YGa_2_O_7.5_ is slightly higher than those of other samples, which we believe is due to a retained proton contribution.^16^ Oxide ion conductivity seems to dominate in *x* = 0.15 and 0.3 compositions at high temperatures, as their conductivities are invariant in wet and dry atmospheres above ∼850 °C. The *x* = 0.6, 1.0, and 1.5 materials have a primitive monoclinic structure, but with a monoclinic angle close to 90°, and display similar conductivities. At the highest temperature, their conductivities under air and dry N_2_ converge, suggesting oxide conductivity dominates. At lower temperatures, a significant proton contribution is observed (Figure 9c). This is maximized at approximately 350–500 °C, where there is an optimum balance of water content and proton mobility, and then decreases as the temperature increases due to water loss. Changes in conductivity at the *P* to *C* phase transition are significantly smaller than for *x* ≤ 0.3 samples. Only changes in the gradient of log(σ) against 1/*T* are observed, reflecting changes in activation energy. This is consistent with the smaller structural changes observed at the phase transition by PXRD. In particular, there is no abrupt cell volume increase for these compositions (see Figure S11).

The remaining *x* ≥ 2 compositions exhibit slightly different behavior, in line with the change to a *C*2 structure. They have higher activation energies for ionic migration at all temperatures than the *x* < 2 compositions. The small low-temperature proton contribution to conductivity decreases with an increase in Sr content, consistent with the diminishing tendency to incorporate water. The conductivity of the *x* = 2 composition, with the *C*2 structure (90°), is higher than the others, which all have comparable conductivities at 1000 °C. This difference cannot be accounted for by the pellet density difference (90% vs 88%) and is therefore likely to be structural in origin. Compositions close to the boundary between two distinct structures often have unusual microstructures, which can influence the properties.

Figure 9 also summarizes conductivity properties as a function of composition across the series. It shows that conductivity decreases slightly with Sr content at both high and low temperatures. This is consistent with the decreasing free volume in the unit cell, resulting in less room for ions to migrate. An approximate proton transport number, *t*_H_, was calculated at 600 and 1000 °C for each composition using
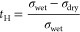
where σ_wet_ and σ_dry_ are the measured conductivities in wet and dry atmospheres, respectively. This assumes dominant ionic conduction under both conditions. The results are plotted in Figure 9b. The proton contribution at 600 °C increases to a maximum in the middle of the series where the structures are metrically orthorhombic before decreasing again to significantly lower values in Sr-rich phases, consistent with the limited tendency of Sr_3_YGa_2_O_7.5_ to incorporate water. At 1000 °C, the proton transport numbers are much lower, and oxide ion conductivity dominates. Ba_3_YGa_2_O_7.5_ is an exception with an approximate half-order-of-magnitude increase in conductivity in air across the whole measured temperature range. As discussed elsewhere,^16^ this suggests a proton contribution remains at high temperatures.

### Structure–Property Relationships

The Ba_3–*x*_Sr_*x*_YGa_2_O_7.5_ system exhibits a rich structural chemistry as a function of both composition and temperature that correlates strongly with ionic conductivity. We can rationalize many of the changes on the basis of the different bonding requirements of Ba and Sr. From Figure 4e, it is clear that the smaller Sr ion leads to large rotations of octahedra and tetrahedra in the (YO_6/2_)(GaO_4/2_) slabs relative to the untilted *Cmcm* aristotype. This is analogous to the octahedral tilting seen in ABO_3_ perovskites to optimize bonding of the ≤12-coordinate A site. In the A_3_OhTd_2_O_7.5_ structure, the maximum A site coordination numbers differ due to the oxygen deficiency relative to perovskite and are summarized for the four unique A sites in Table 2. Taking site multiplicities for Ba1–4 into account (2*e*, 2*f*, 4*g*, and 4*g* Wyckoff positions), we find the average A site coordination number is 10, as expected for an ABO_2.5_ composition relative to 12-coordinate A in an ABO_3_ perovskite. Because assigning formal coordination numbers and average bond lengths for Ba and Sr is somewhat arbitrary, Table 2 also contains the number of bonds that make up >97.5% of the bond valence sum for each A, and the average of those bond lengths. In the case of Ba1, for example, although 10 oxygens could potentially form part of its coordination sphere, there is a significant break between the eight nearer oxygens (<3.13 Å) and the final two (>3.64 Å), which contribute only 0.05 to the bond valence sum. The 97.5% cutoff allows us to focus on the more dominant interactions.

**Table 2:** A Site Coordination Numbers, Bond Distances, and Bond Valence Sums in A_3_YGa_2_O_7.5_ Phases

			Ba–O			Sr–O			Ba to Sr
	site	CN (Ba/Sr)	average (Å)	variance (Å^2^)	BVS	average (Å)	variance (Å^2^)	BVS	no. of longer bonds
all A–O	Ba1/Sr3	10/10	3.012	0.130	1.91	2.896	0.173	1.93	3
	Ba2/Sr4	8/8	2.858	0.011	1.76	2.722	0.033	1.73	0
	Ba3/Sr2	11/11	3.050	0.089	1.72	2.987	0.177	1.57	4
	Ba4/Sr1	10/10	2.961	0.038	1.87	2.891	0.121	1.75	2
97.5% BVS	Ba1/Sr3	8/8	2.855	0.027	1.86	2.744	0.091	1.89	3
	Ba2/Sr4	8/8	2.858	0.011	1.76	2.722	0.033	1.73	0
	Ba3/Sr2	9/9	2.925	0.012	1.66	2.818	0.043	1.54	1
	Ba4/Sr1^a^	10/9	2.961	0.038	1.87	2.812	0.121	1.73	2

a For Sr1, the standard deviation is calculated over the 10 bond distances equivalent to that of Ba4. The final column gives the number of bond lengths that are longer for A = Sr.

We see from Table 2 that sites Ba3 and Ba4 in the (YO_6/2_)(GaO_4/2_) slabs have higher coordination numbers and correspondingly longer average bond lengths compared to those of Ba1 and Ba2. We also see that the polyhedral tilting upon introduction of the smaller Sr leads to more distorted A site coordination environments. This is shown by the variance of the important Sr–O bond distances being approximately 3 times that of Ba–O for each site. It is interesting to note that for all sites except the strictly eight-coordinate A2, several A–O distances increase upon introduction of the smaller Sr^2+^. This is the corollary of the bond valence distortion theorem discussed by Brown,^49^ i.e., that coordination environments distort to produce shorter and longer bond lengths to increase bond valence sums to expected values when a cation is too small for a given cavity. All of these observations are consistent with the general trend for Sr^2+^ to exhibit coordination numbers lower than those of Ba^2+^ in oxides. Waroquier’s analysis, for example, shows ∼21% of Sr^2+^ coordination environments can be described as eight-coordinate compared to only 9% for Ba^2+^.^50^

The lower effective coordination numbers of site Ba1 and (especially) Ba2 then imply a tendency for Sr segregation on these positions, consistent with the HAADF-STEM observations. This helps explain the structural evolution in Figure 3. As the Sr content is increased from *x* = 0 to 0.6, the tilt pattern and *P*2/*c* structure of Ba_3_YGa_2_O_7.5_ are initially retained, though with a decreasing β angle as the Sr content increases. As the Sr content increases further, partial segregation of Sr to the Ga_2_O_7_ layers will create regions of Sr-like stacking in the Ba structure, with the microdomain structure leading to a metrically orthorhombic unit cell. At the Ba_1.5_Sr_1.5_ composition, these Sr-like layers are clearly visible in the HAADF-STEM images, even though the structure remains primitive. By Ba_1.0_Sr_2.0_, the smaller average A size favors the centered structure, and the lack of defects observed by HAADF-STEM is consistent with the Ga_2_O_7_ layers having a consistent Sr-rich occupancy. The increasing relative stability of the centered structure with *x* leads to an initial decrease in the phase transition temperature upon heating of low-*x* samples plotted in Figure 3, followed by an increase as the Sr content increases toward *x* = 3. A similar decrease in the phase transition temperature was reported by Abakumov et al. as the size of Oh cations was increased in Ba_3_OhTd_2_O_7.5_ (Oh = lanthanoid, and Td = Al or Ga) compounds.^17^

The observed evolution of conductivity across the series can be related to these structural changes. The arrangement of octahedra, tetrahedra, and vacant oxygen sites in the structure (see Figure 4) suggests a potential low-energy pathway for O^2–^ migration involving reorientation of Ga_2_O_7_ tetrahedra. High-temperature diffraction experiments show phase transitions associated with either reorientation of these tetrahedra (at low *x*) or their local disorder, and conductivity data show changes at the corresponding temperatures. For *x* = 0, 0.15, and 0.3, we see abrupt changes in conductivity at *T*_c_ related to abrupt volume changes. For *x* > 0.3, the unit cell volume changes continuously at *T*_c_, and the conductivity changes more gradually. The importance of the structure of the Ga_2_O_7_ layer to conductivity is further highlighted by the increase in the ion migration activation energy at *x* = 2, where the structure changes from primitive to centered, which is associated with local changes in the Ga_2_O_7_ environment. The larger cell volume of Ba-rich samples also allows significant water uptake leading to a large proton conductivity contribution that is largest around 350–500 °C, before H_2_O is lost at higher temperatures.

## Conclusions

In conclusion, single-phase materials can be prepared across the whole Ba_3–*x*_Sr_*x*_YGa_2_O_7.5_ solid solution range and exhibit a complex structural phase diagram as a function of composition and temperature. Compounds at the Ba- and Sr-rich compositional extremes have *P*2/*c* and *C*2 structures, respectively, at room temperature. As the Sr content is increased from *x* ≈ 0 to compositions such as Ba_2.4_Sr_0.6_YGa_2_O_7.5_ and Ba_1.5_Sr_1.5_YGa_2_O_7.5_, HAADF-STEM imaging reveals intergrowths of Sr-like regions in the Ba structure type. The resultant microstructure leads to a metrically orthorhombic unit cell. For *x* = 2 Ba_1_Sr_2_YGa_2_O_7.5_, diffraction data, STEM imaging, and SHG measurements suggest a structure in space group *C*2 with a ∼90° monoclinic angle. Most of the compounds in the series appear to undergo a transition to a higher-symmetry, disordered *Cmcm*-like structure at high temperatures, except for Ba_3_YGa_2_O_7.5_, which adopts an alternative Ga_2_O_7_ ordering pattern, and the *x* > 2 Sr-rich compositions where no phase transition is observed up to 1000 °C.

Impedance spectroscopy shows that all members of the family show either oxide ion or proton conductivity, depending on the experimental conditions. There is a general decrease in conductivity as the Sr content increases, consistent with the smaller unit cell volumes. Conductivity properties evolve smoothly from *x* = 0 to *x* = 1.5. From *x* = 2, there is an abrupt increase in the activation energy for ion migration, corresponding to the structural change to *C*2. The contribution of protons to conductivity peaks in the middle of the series before decreasing as the Sr content increases. Unlike the case for Ba_3_YGa_2_O_7.5_, TGA and proton NMR experiments showed Sr_3_YGa_2_O_7.5_ takes up very little water and is stable in moist atmospheres. The Sr-rich compounds are therefore of particular interest for further development as solid electrolyte materials.

## Supporting Information Available

Plots of cell parameters for all compositions, Rietveld plots for all compositions, decrease of the symmetry group–subgroup graph for *x* = 1.5, Rietveld agreement factors for 64 subgroup models, crystallographic data for BaSr_2_YGa_2_O_7.5_ and Sr_3_YGa_2_O_7.5_, temperature dependence of unit cell parameters for all compositions, dependence of transition temperatures on hydration, variable-temperature powder diffraction data, thermal expansion coefficients, variable-temperature diffraction data of Ba_2.85_Sr_0.15_YGa_2_O_7.5_, TGA data for representative compositions, and ^1^H NMR data of moisture-exposed samples (PDF)
Animation of the structural changes in ISOVIZ format (AVI)

## Supplementary Material

cm1c04372_si_001.pdf

cm1c04372_si_002.avi
