# Supplementary material for: Oxide Ion Conductivity, Proton Conductivity, and Phase Transitions in Perovskite-Derived Ba3–xSrxYGa2O7.5 0 ≤ x ≤ 3 Materials
Source: Chem Mater. 2022 Mar 28;34(7):3185–96. doi: 10.1021/acs.chemmater.1c04372 (PMC9086979; doi:10.1021/acs.chemmater.1c04372)
Supplement: Supplementary file 1 — cm1c04372_si_001.pdf [file cm1c04372_si_001.pdf]

# Oxide ion conductivity, proton conductivity and phase transitions in perovskite-derived $Ba_{3-x}Sr_xYGa_2O_{7.5}$ $0 \leq x \leq 3$ materials

Chloe A. Fuller[†,††], James I. Murrell[†,†††], Douglas A. Blom[‡], Thomas Vogt[‡‡], Weiguo Zhang,[‡‡‡] P. Shiv Halasyamani[‡‡‡], Ivana Radosavljevic Evans[†], and John S. O. Evans[†,*]

† Department of Chemistry, Durham University, Science Site, South Road, Durham DH1 3LE, United Kingdom

‡ Department of Chemical Engineering and NanoCenter, University of South Carolina, Columbia, SC 29208, USA

†† Department of Chemical Engineering, Chemistry and Biochemistry and NanoCenter, University of South Carolina, Columbia, SC 29208, USA

‡‡‡Department of Chemistry, University of Houston, Texas 77204-5003, USA

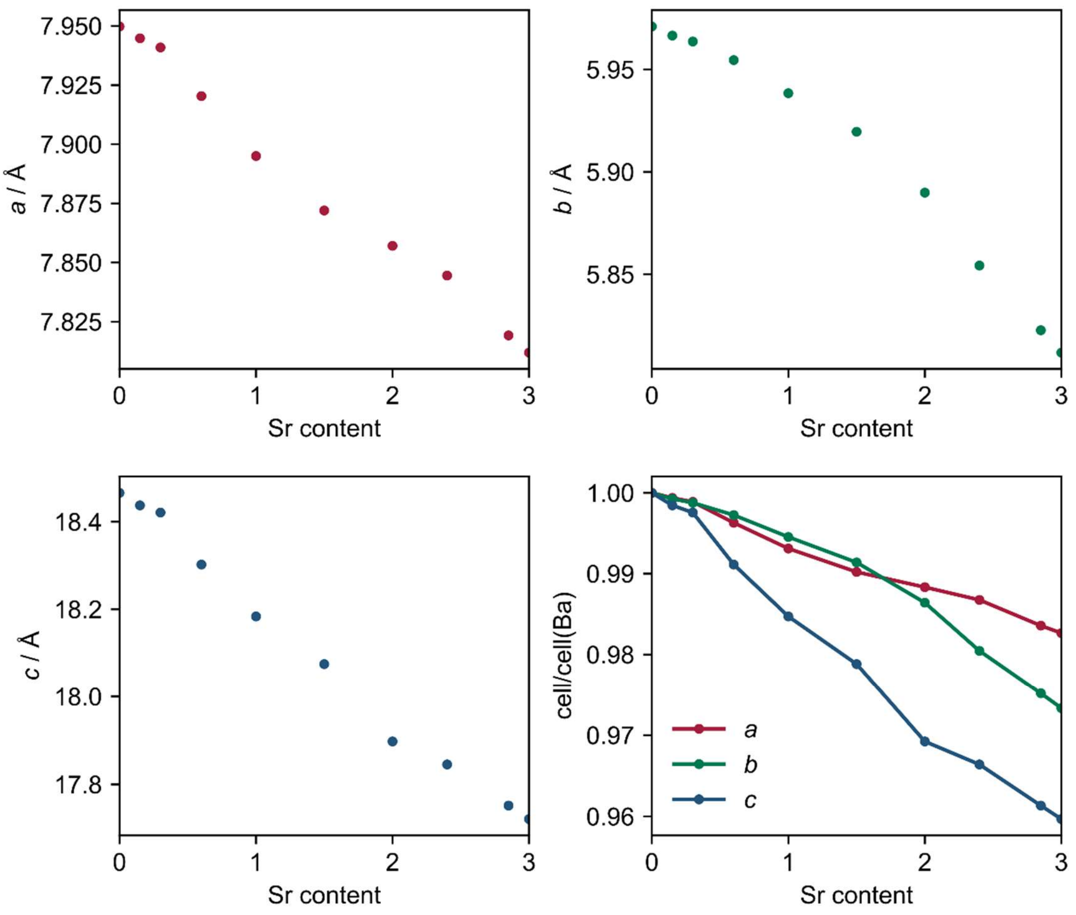


*Figure S1. Cell parameter trends across the $Ba_{3-x}Sr_xYGa_2O_{7.5}$ series.*

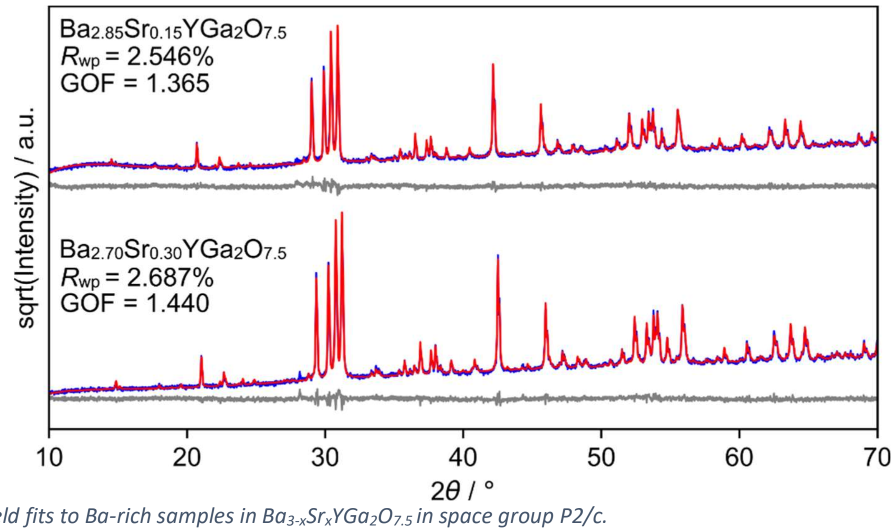


*Figure S2. Rietveld fits to Ba-rich samples in $Ba_{3-x}Sr_xYGa_2O_{7.5}$ in space group P2/c.*

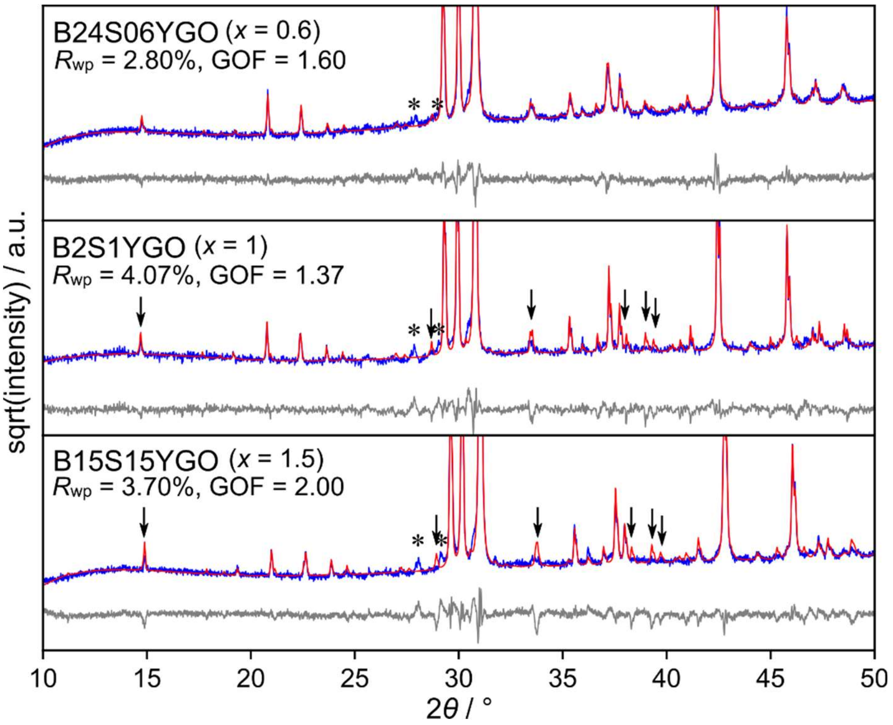


*Figure S3. Rietveld fits to the PXRD patterns of the labelled compounds in $Ba_{3-x}Sr_xYGa_2O_{7.5}$. Only a zoomed section of the whole pattern is shown and the intensities are on a square root scale to exaggerate small features. Asterisks indicate minor impurity peaks and arrows highlight areas where there is significant over-calculation of the intensity.*

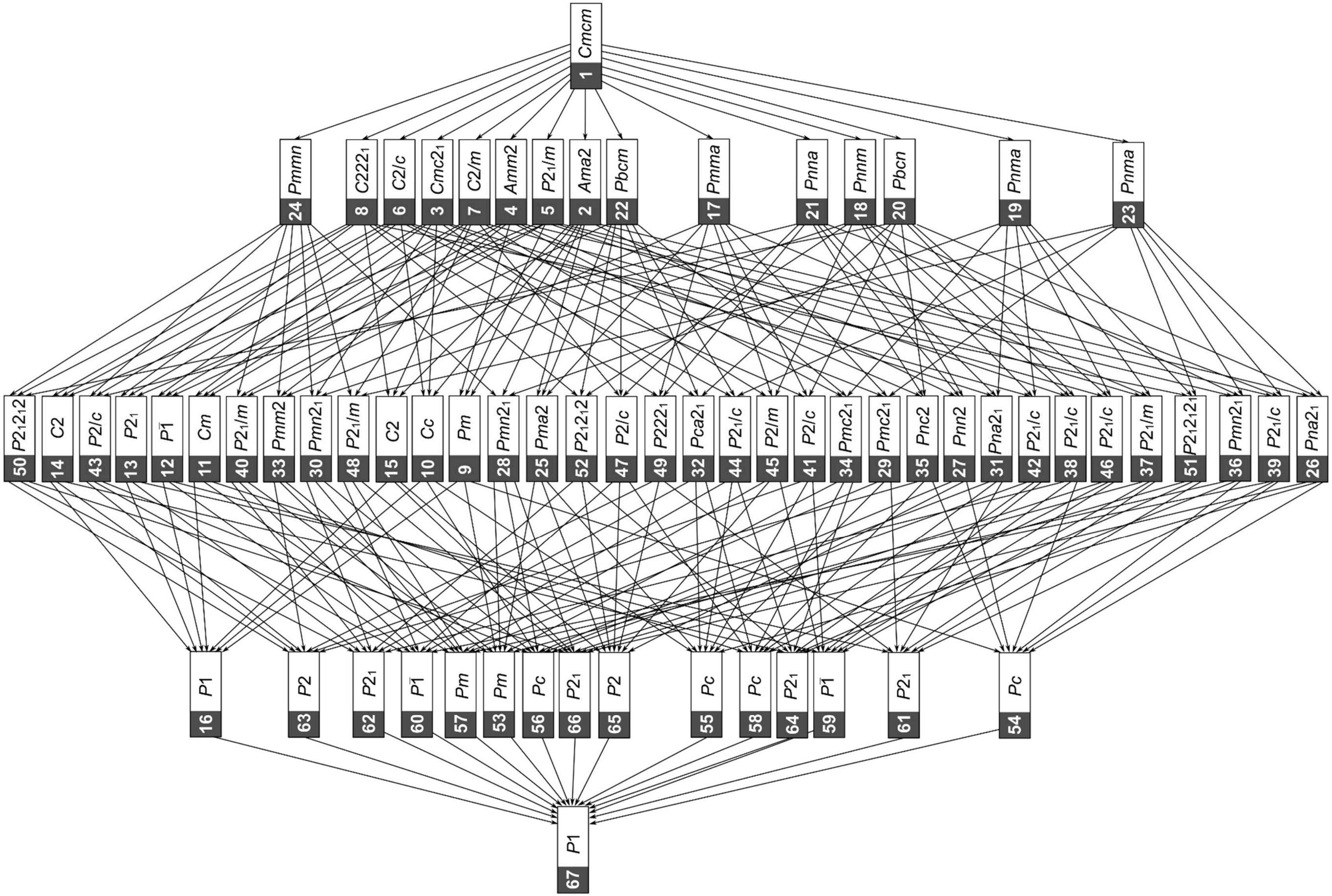


*Figure S4. Subgroup tree from parent Cmcm to child P1. White numbers represent the subgroup serial number used in the symmetry descent Rietveld analysis.*

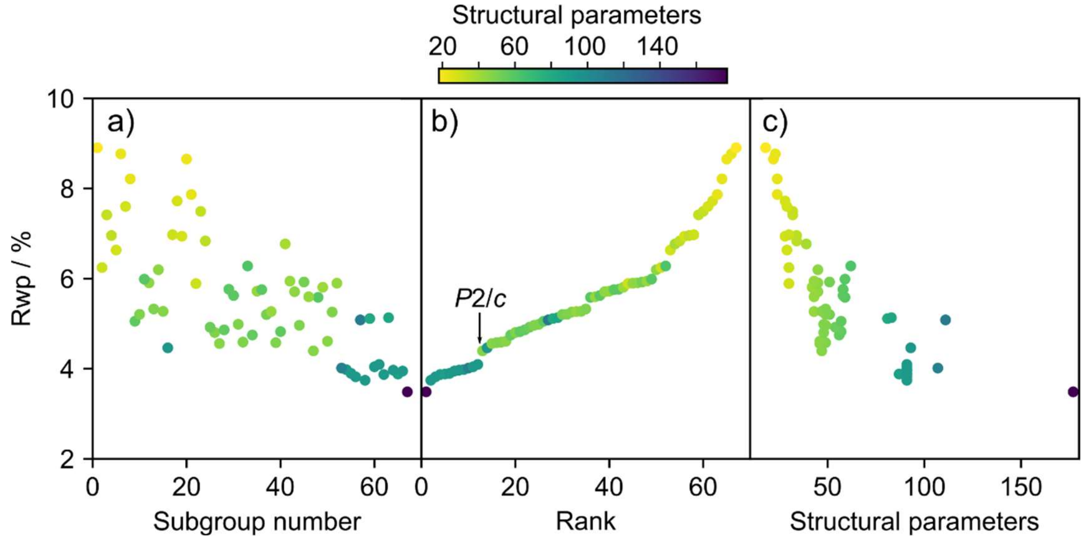


*Figure S5. Summary of the results of the symmetry descent Rietveld analysis. Each point corresponds to a Rietveld refinement in a different subgroup. The subgroup serial numbers correspond to those given in Figure S4. The blue point is the P1 child base. Points are colour coded according to the number of free structural parameters refined in each subgroup.*

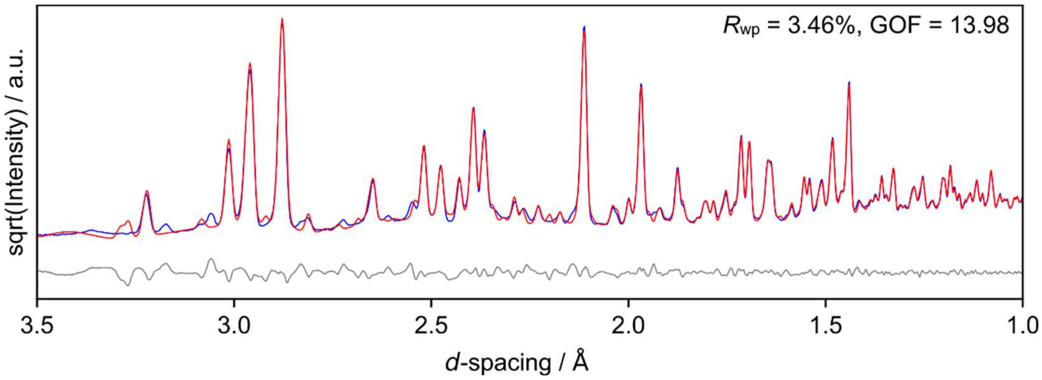


*Figure S6. Rietveld fit of neutron bank 4 diffraction data of $Ba_{1.5}Sr_{1.5}YGa_2O_{7.5}$ in P1 (subgroup 67).*

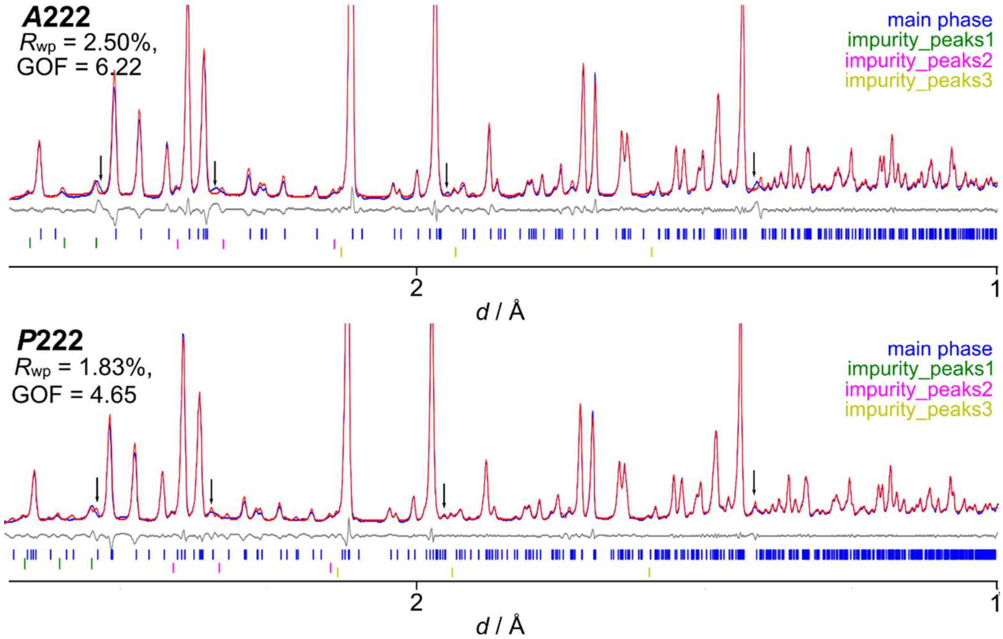


*Figure S7. Neutron diffraction patterns of $Ba_{1.5}Sr_{1.5}YGa_2O_{7.5}$ Pawley fitted in two different space groups. This corresponds to the main phase in the legend and the blue tick marks. Peaks due to minor impurities are fitted with free intensities so that the differences between P and A are emphasised; their positions are shown with green, pink and yellow ticks. Arrows indicate primitive peaks which are predicted in P222 but not A222.*

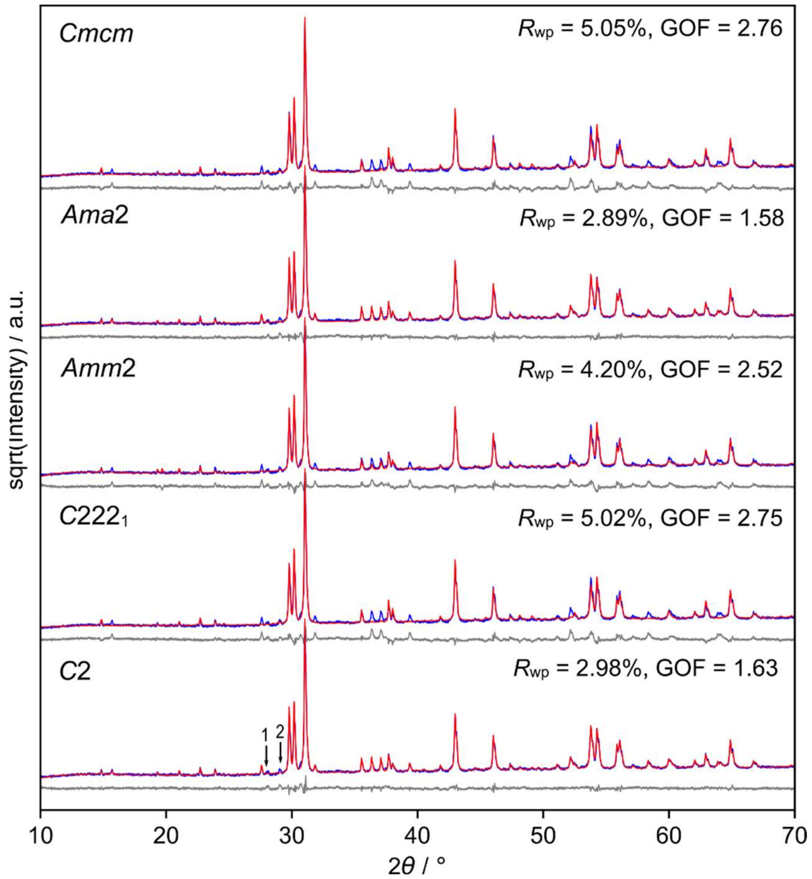


*Figure S8. Rietveld fits to the PXRD data of $BaSr_2YGa_2O_{7.5}$ in various space groups. Numbers 1 and 2 indicate peaks that are not predicted by space groups Ama2 and C2*

*Table S1. Crystallographic data for the room-temperature structure of $BaSr_2YGa_2O_{7.5}$ with unit cell parameters a = 17.8969(2) Å, b = 5.8899(1) Å, c = 7.8572(1) Å, β = 90.014(4)° in space group C2. Due to the lack of neutron data on this composition, only the cation positions were refined and the anion positions are fixed at the values obtained from structural refinements on the isostructural $Sr_3YGa_2O_{7.5}$ (see later). $B_{iso}$ values were constrained to be equal for the same atom types.*

| Atom | Site | *x*/*a* | *y*/*b* | *z*/*c* | $B_{iso}$ / Å$^2$ |
|---|---|---|---|---|---|
| Ba/Sr1 | 2*a* | 0 | 0.063(3) | 0 | 1.99(4) |
| Ba/Sr2 | 2*a* | 0 | 0.080(3) | 0.5 | 1.99(4) |
| Ba/Sr3 | 4*c* | 0.1683(5) | 0.488(3) | 0.9861(12) | 1.99(4) |
| Ba/Sr4 | 4*c* | 0.1656(5) | 0.501(3) | 0.5216(11) | 1.99(4) |
| Y1 | 4*c* | 0.3428(3) | 0.507(2) | 0.746(2) | 0.66(9) |
| Ga1 | 4*c* | 0.3119(3) | 0.490(3) | 0.255(2) | 1.56(9) |
| Ga2 | 4*c* | 0.0139(4) | 0.541(2) | 0.255(2) | 1.56(9) |
| O1 | 2*a* | 0 | 0.5063 | 0 | 2.37(15) |
| O2 | 4*c* | 0.36436 | 0.5009 | 0.0421 | 2.37(15) |
| O3 | 4*c* | 0.33815 | 0.4837 | 0.4699 | 2.37(15) |
| O4 | 4*c* | 0.09053 | 0.3282 | 0.2645 | 2.37(15) |
| O5 | 4*c* | 0.04112 | 0.8321 | 0.2708 | 2.37(15) |
| O6 | 4*c* | 0.26255 | 0.2008 | 0.2287 | 2.37(15) |
| O7 | 4*c* | 0.23473 | 0.6981 | 0.2232 | 2.37(15) |
| O8 | 4*c* | 0.92642 | 0.4116 | 0.3129 | 2.37(15) |

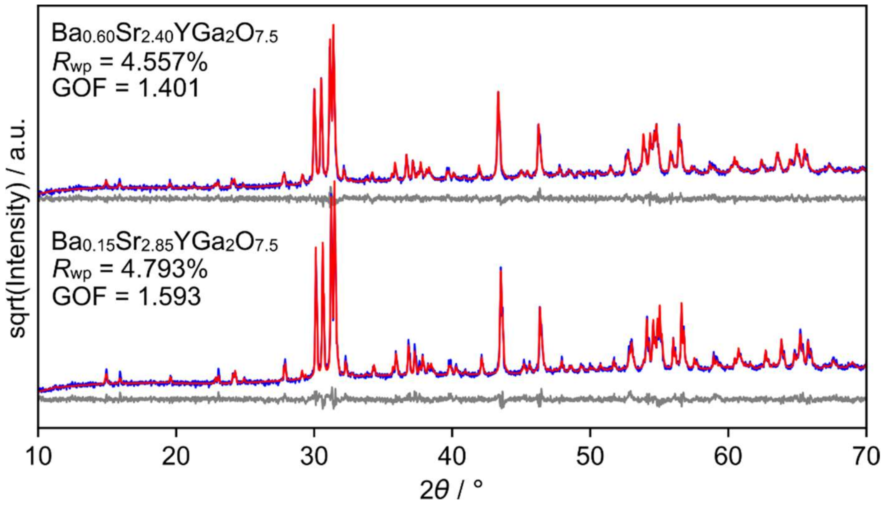


*Figure S9. Rietveld fits to Sr-rich samples in $Ba_{3-x}Sr_xYGa_2O_{7.5}$ in space group C2.*

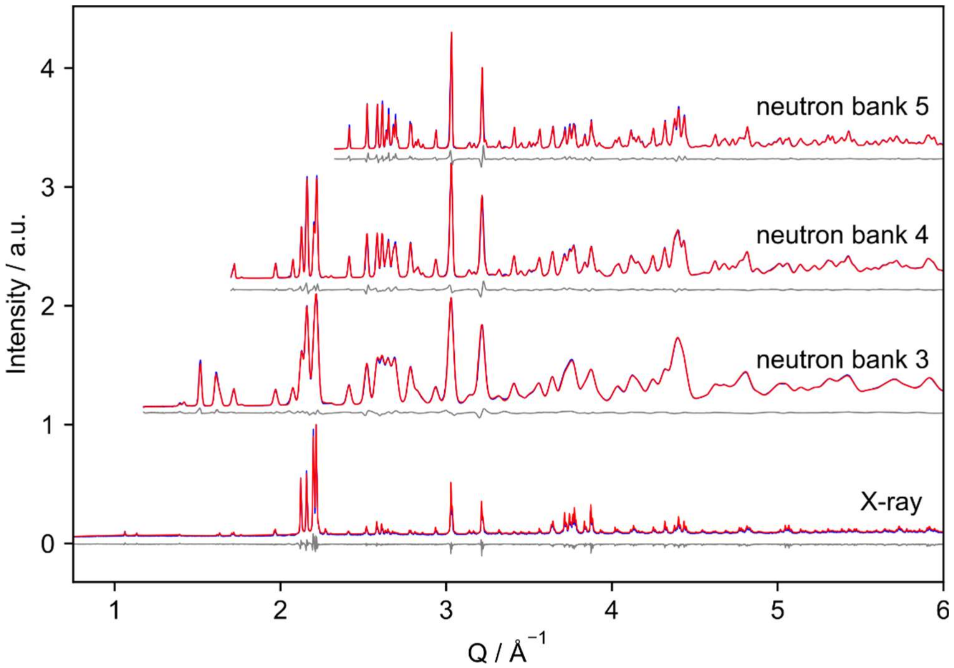


*Figure S10. Joint X-ray and neutron Rietveld refinement of $Sr_3YGa_2O_{7.5}$. The top 3 datasets are from the 3 highest resolution banks on POLARIS and the bottom set was measured on a laboratory (Cu Kα) diffractometer. Note that each dataset has been normalised to 1 so that they can be shown on the same scale. $R_{wp}$ = 2.69%, GOF = 8.71.*

*Table S2. Crystallographic data for the room temperature structure of $Sr_3YGa_2O_{7.5}$ with unit cell parameters a = 17.7172(2) Å, b = 5.81064(5) Å, c = 7.80650(7) Å and β = 90.6196(6)° in space group C2.*

| Atom | Site | *x*/*a* | *y*/*b* | *z*/*c* | $B_{iso}$ / Å$^2$ |
|---|---|---|---|---|---|
| Sr1 | 2*a* | 0 | 0.0456(4) | 0 | 1.30(5) |
| Sr2 | 2*a* | 0 | 0.1029(4) | 0.5 | 1.02(5) |
| Sr3 | 4*c* | 0.16021(8) | 0.4636(3) | 0.9697(2) | 1.60(5) |
| Sr4 | 4*c* | 0.1752(1) | 0.4854(3) | 0.5024(2) | 1.21(3) |
| Y1 | 4*c* | 0.34320(8) | 0.4925(3) | 0.7565(2) | 0.83(3) |
| Ga1 | 4*c* | 0.3105(1) | 0.4815(3) | 0.2429(2) | 0.53(3) |
| Ga2 | 4*c* | 0.0124(1) | 0.5383(2) | 0.2344(2) | 0.42(3) |
| O1 | 2*a* | 0 | 0.5090(5) | 0 | 2.09(8) |
| O2 | 4*c* | 0.3644(1) | 0.5056(4) | 0.0429(3) | 0.96(4) |
| O3 | 4*c* | 0.3383(1) | 0.4812(5) | 0.4704(2) | 1.62(4) |
| O4 | 4*c* | 0.0407(1) | 0.8306(4) | 0.2675(3) | 0.99(4) |
| O5 | 4*c* | 0.0905(1) | 0.3273(3) | 0.2647(4) | 0.86(3) |
| O6 | 4*c* | 0.2623(1) | 0.1998(4) | 0.2270(3) | 0.66(3) |
| O7 | 4*c* | 0.2344(1) | 0.6971(4) | 0.2258(3) | 0.99(4) |
| O8 | 4*c* | 0.9264(1) | 0.4097(4) | 0.3135(3) | 0.94(3) |

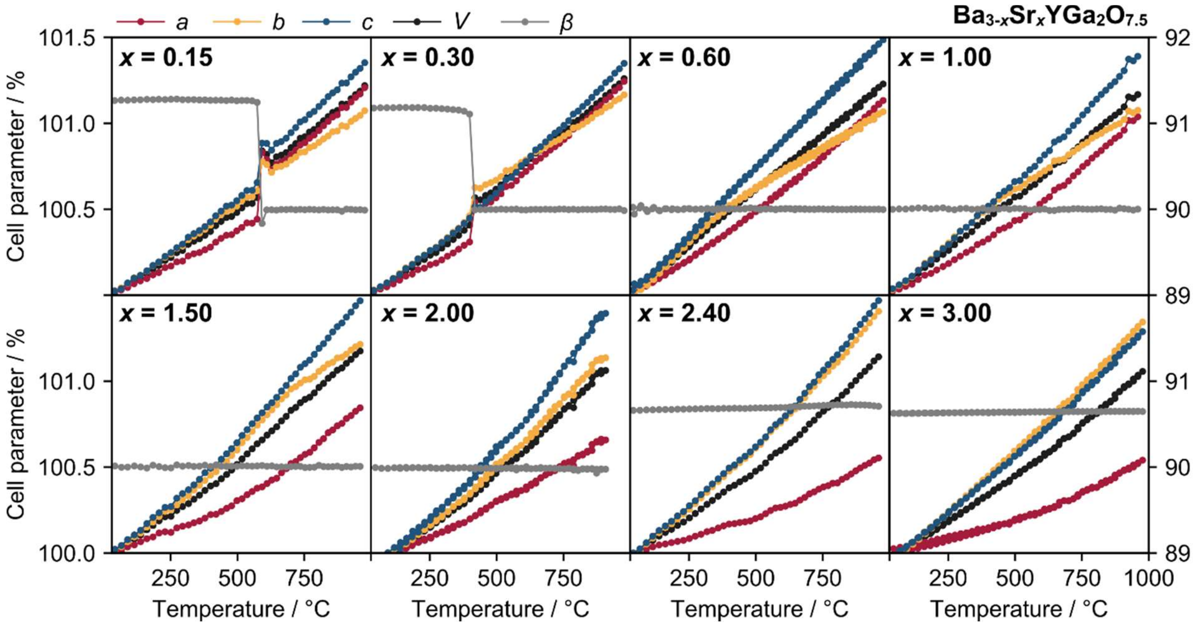


*Figure S11. Percentage changes in cell parameters extracted from variable temperature x-ray powder diffraction on the $Ba_{3-x}Sr_xYGa_2O_{7.5}$ series. Volume changes have been divided by three so they can be plotted on a comparable y-axis range to a, b and c [note that for a cubic material linear thermal expansion is 1/3 of the volume thermal expansion].*

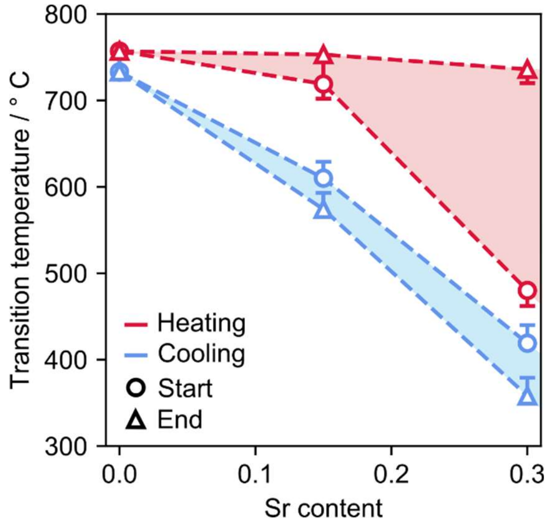


*Figure S12. Hydration of the compounds affects the variable temperature behavior and causes significant hysteresis in the phase transition temperature of Ba-rich compounds. If the sample is dried prior to the VT experiment, this hysteresis is eliminated. Plot shows transition temperatures for $Ba_{3-x}Sr_xYGa_2O_{7.5}$ with $x \leq 0.3$. Circle markers denote the onset of the phase transition and triangles indicate the temperature at which the phase transition is complete. Red and blue colours show heating and cooling cycles respectively, and shaded areas show two-phase regions. Vertical error bars represent the uncertainty associated with measuring the diffraction pattern in discrete temperature intervals (every 20–50 °C).*

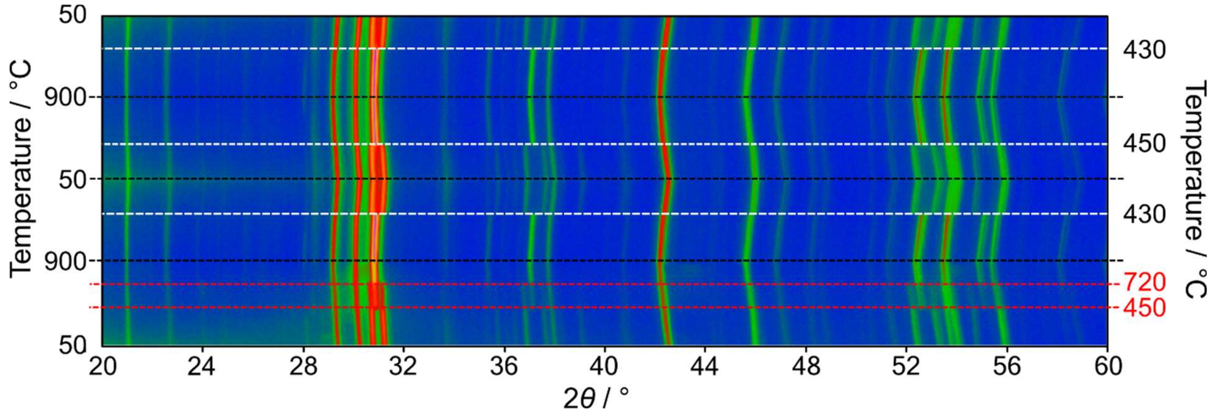


*Figure S13. Artificially colour coded surface plot of diffraction patterns from variable temperature cycling experiments on a sample of $Ba_{2.7}Sr_{0.3}YGa_2O_{7.5}$ which had been exposed to moisture. The moist sample was warmed from 50 to 900 °C, cooled to 50 °C, warmed to 900 °C then finally cooled to 50 °C. Black dotted lines show the changes from heating to cooling. Red dotted lines enclose the two-phase region on initial cooling where dehydration occurs. White lines highlight phase transitions on subsequent cycles. Temperatures of important transitions are given on the right-hand side. Colour coding is blue to red for low to high intensity. The total experimental time was approximately 4 days.*

*Table S3. Volume thermal expansion coefficients between room temperature and 1000°C for $Ba_{3-x}Sr_xYGa_2O_{7.5}$, above and below the phase transition where necessary.*

| Sr content | $\alpha_v$ = 1/V (ΔV/ΔT) / $10^{-5}$ $K^{-1}$ | |
|---|---|---|
| | **Below** | **Above** |
| 0.00 | 3.31(3) | 3.63(5) |
| 0.15 | 3.11(3) | 3.43(13) |
| 0.30 | 3.14(5) | 3.87(3) |
| 0.60 | 3.91(6) | 3.82(2) |
| 1.00 | 3.61(4) | 3.94(4) |
| 1.50 | 3.66(7) | 4.10(5) |
| 2.00 | 3.95(5) | 5.1(4) |
| 2.40 | 4.14(15) | - |
| 3.00 | 3.50(3) | - |

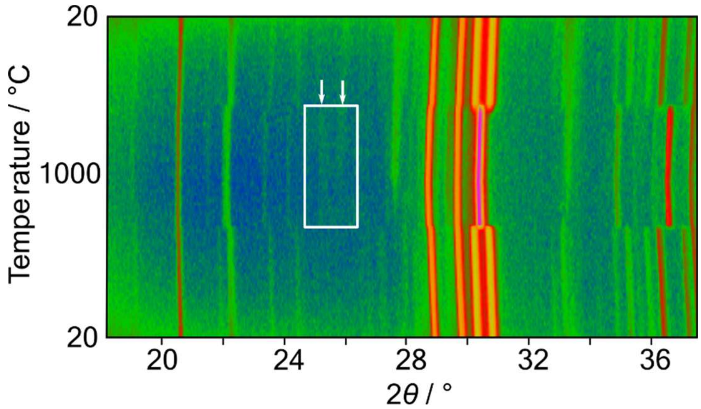


*Figure S14. Artificially colour coded surface plot of diffraction patterns of $Ba_{2.85}Sr_{0.15}YGa_2O_{7.5}$ measured on heating and cooling between room temperature and 1000 °C. The white box highlights the diffuse scattering seen around the positions of the Tamazyan-type ordering peaks seen in $Ba_3YGa_2O_{7.5}$,[1] indicated by the arrows.*

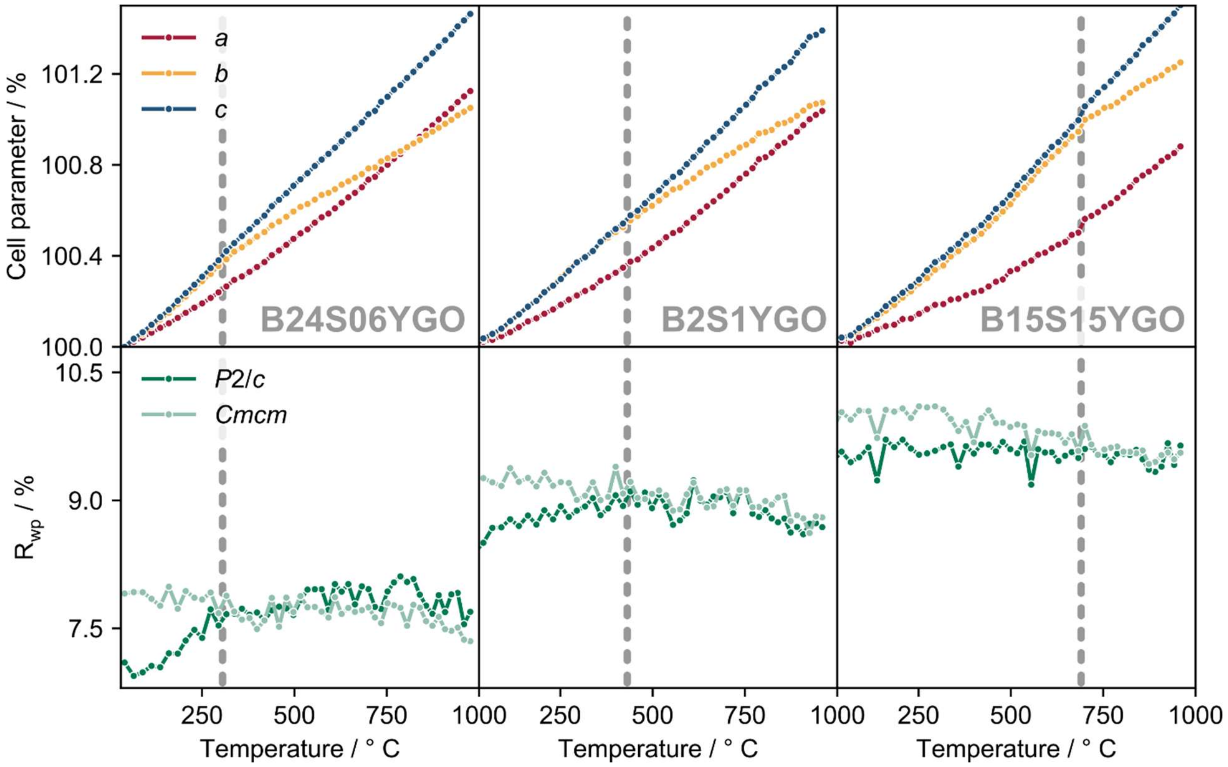


*Figure S15. Percentage change in cell parameters for three compositions (x = 0.6, 1.0 and 1.5) as a function of temperature and the $R_{wp}$ obtained when using structural models in P2/c and Cmcm. Dashed grey lines indicate the temperature of the phase transition. Above this temperature the two space groups give essentially identical R-factors.*

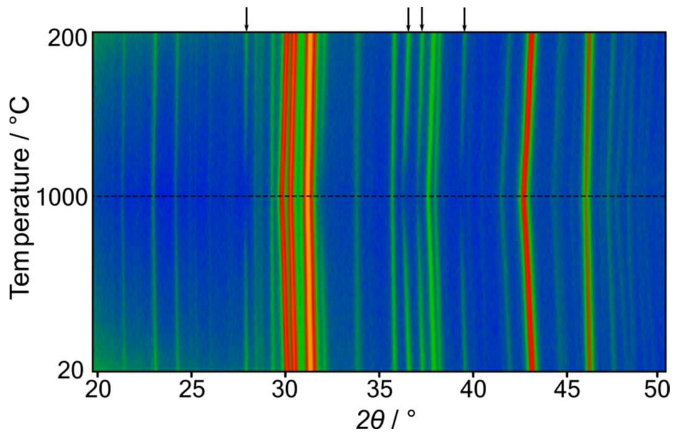


*Figure S16 Variable temperature diffraction data of $BaSr_2YGa_2O_{7.5}$ on heating and cooling between room temperature and 1000 °C. Arrows above the figure show peaks which undergo significant intensity changes at the phase transition*.

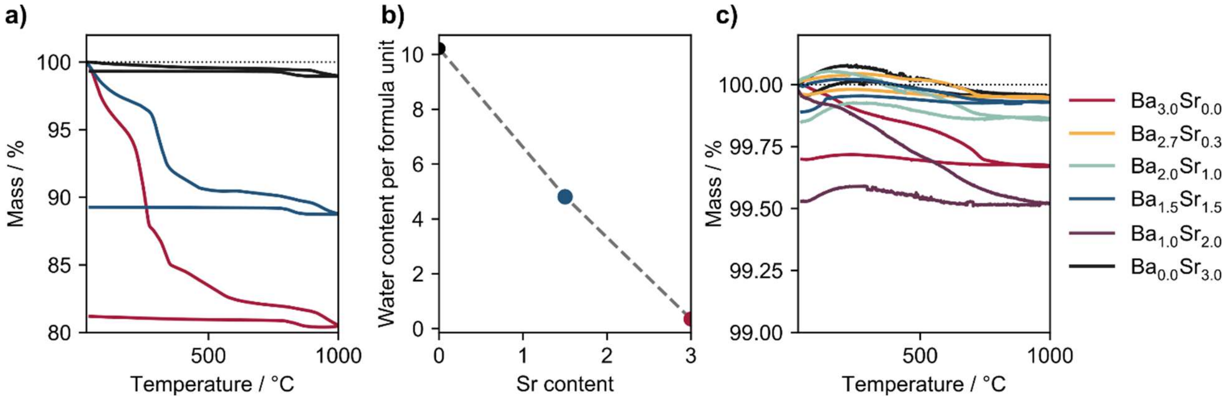


*Figure S17. a) TGA traces of $Ba_3YGa_2O_{7.5}$, $Ba_{1.5}Sr_{1.5}YGa_2O_{7.5}$ and $Sr_3YGa_2O_{7.5}$ after deliberate exposure to moist atmosphere. b) Water content per formula unit based on the assumption that all mass loss in a) is the result of water loss. c) TGA traces of $Ba_{3-x}Sr_xYGa_2O_{7.5}$ compounds immediately following heating at 1150 °C for 8.5 h then cooling at 5 °C min$^{-1}$ to 150 °C. Water uptake/loss depends strongly on the sample history. Minor increases in mass above 100% at low temperature on heating and cooling in panel c) are caused by buoyancy effects; similar "humps" are observed with blank sample holders.*

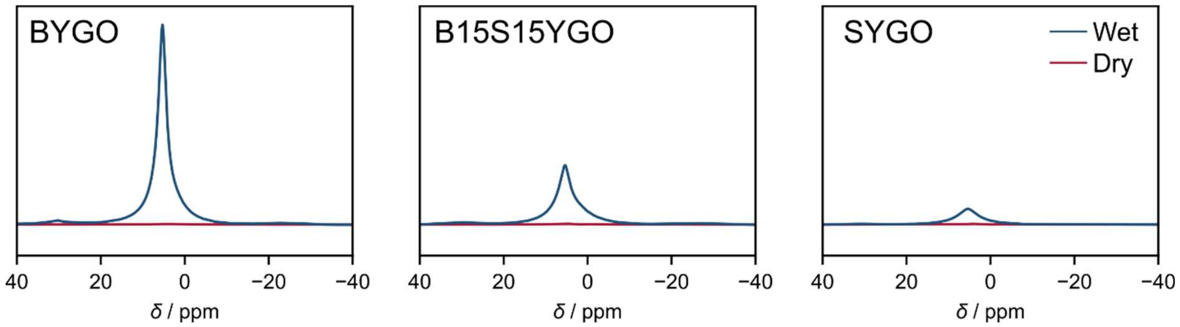


*Figure S18. Proton NMR spectra of selected $Ba_{3-x}Sr_xYGa_2O_{7.5}$ compounds after drying and after moisture chamber treatment.*

## REFERENCES

1. Fuller, C. A.; Blom, D. A.; Vogt, T.; Evans, I. R.; Evans, J. S. O., Oxide and proton conductivity in a family of highly oxygen deficient perovskite derivatives. *Journal of the American Chemical Society* **2021**, accepted for publication.
